# Preclinical Development of T Cells Engineered to Express a T-Cell Antigen Coupler Targeting Claudin 18.2–Positive Solid Tumors

**DOI:** 10.1158/2326-6066.CIR-24-0138

**Published:** 2024-10-15

**Authors:** Stacey X. Xu, Ling Wang, Philbert Ip, Ritu R. Randhawa, Tania Benatar, Suzanna L. Prosser, Prabha Lal, Alima Naim Khan, Thanyashanthi Nitya-Nootan, Gargi Thakor, Heather MacGregor, Danielle L Hayes, Andrea Vucicevic, Princy Mathew, Sadhak Sengupta, Christopher W. Helsen, Andreas G. Bader

**Affiliations:** 1 Triumvira Immunologics, Inc., headquartered in Austin, Texas, with research facilities in Hamilton, Canada.

## Abstract

The T-cell antigen coupler (TAC) is a chimeric receptor that facilitates tumor antigen–specific activation of T cells by co-opting the endogenous T-cell receptor complex in the absence of tonic signaling. Previous data demonstrate that the TAC affords T cells with the ability to induce durable and safe antitumor responses in preclinical models of hematologic and solid tumors. In this study, we describe the preclinical pharmacology and safety of an autologous Claudin 18.2 (CLDN18.2)-directed TAC T-cell therapy, TAC01-CLDN18.2, in preparation for a phase I/II clinical study in subjects with CLDN18.2-positive solid tumors. Following a screen of putative TAC constructs, the specificity, activity, and cytotoxicity of TAC T cells expressing the final CLDN18.2-TAC receptor were evaluated *in vitro* and *in vivo* using gastric, gastroesophageal, and pancreatic tumor models as well as human cells derived from normal tissues. CLDN18.2-specific activity and cytotoxicity of CLDN18.2-TAC T cells were observed in coculture with various 2D tumor cultures naturally expressing CLDN18.2 as well as tumor spheroids. These effects occurred in models with low antigen levels and were positively associated with increasing CLDN18.2 expression. CLDN18.2-TAC T cells effectively eradicated established tumor xenografts in mice in the absence of observed off-target or on-target/off-tumor effects, elicited durable efficacy in recursive killing and tumor rechallenge experiments, and remained unreactive in coculture with human cells representing vital organs. Thus, the data demonstrate that CLDN18.2-TAC T cells can induce a specific and long-lasting antitumor response in various CLDN18.2-positive solid tumor models without notable TAC-dependent toxicities, supporting the clinical development of TAC01-CLDN18.2.

## Teaser

The authors report the preclinical development of TAC01-CLDN18.2 for the treatment of Claudin 18.2-positive solid tumors. TAC01-CLDN18.2 is an autologous T-cell therapy that capitalizes on signaling through the endogenous TCR for efficacious and safe antitumor responses.

## Introduction

Cell therapy, particularly the adoptive cell transfer of T cells genetically engineered with chimeric antigen receptors (CAR), illustrated the possibility of curative outcomes in hematologic B-cell malignancies and set an expectation for similar successes in other tumor types. However, thus far, CAR T-cell therapies have failed to show high efficacy against solid tumors, presumably due to the more complex nature of solid tumors compared with hematologic malignancies likely demanding a more durable T-cell approach. To address this, the T-cell antigen coupler (TAC), a novel chimeric receptor, was designed to maximize the therapeutic potential of T cells (1). It facilitates the MHC-unrestricted redirection of T cells to tumor cells via an antigen-binding domain and activates T cells by co-opting the natural T-cell receptor (TCR) complex via a CD3ε-binding domain fused to the transmembrane and cytosolic domains of the CD4 co-receptor. Thus, the TAC cannot signal on its own and, rather, hijacks the entire TCR and its canonical downstream pathways in an antigen-specific manner. In a head-to-head comparison with an analogous, second-generation CAR, TAC T cells led to more efficacious and safer outcomes in tumor mouse models (1). Key advantages of TAC T cells are a lack of tonic signaling and premature exhaustion in the absence of antigen recognition and—upon target engagement—the formation of a normal immune synapse, the secretion of relatively low cytokine levels, and persistent antitumor activity *in vitro* and *in vivo* (1–3). Accordingly, a phase I clinical study evaluating HER2-TAC T cells in subjects with HER2-positive solid tumors demonstrated promising signs of efficacy and a manageable safety profile, with no immune effector cell–associated neurotoxicity syndrome and only two grade-3 events that resolved with standard of care (4).

Claudin 18.2 (CLDN18.2) belongs to a family of membrane proteins that is comprised of 27 members and has recently emerged as a promising tumor-associated antigen for targeted cancer therapy. Among normal tissues, it is restricted to tight junctions of gastric epithelial cells and involved in the regulation of cell polarity, intercellular adhesion, and exchange of molecules between adjacent cells (5). As a result of oncogenic cell transformation and loss of cell polarity, however, CLDN18.2 expression is no longer confined to tight junctions and is frequently expressed at elevated levels in tumor cells, making it reasonably tumor-selective. CLDN18.2 was found to be overexpressed in Caucasian and Asian patient populations with primary and metastatic gastric cancers (6–12) and was also detected in neoplasia derived from CLDN18.2-negative tissues, such as esophageal, pancreatic, lung, and ovarian cancers (12). To date, promising clinical data have been generated with zolbetuximab, a chimeric IgG1 mAb targeting CLDN18.2 used in patients with advanced or metastatic gastric or gastroesophageal junction cancer (13, 14), as well as CT041, an autologous CLDN18.2-specific CAR T product evaluated in patients with gastrointestinal and metastatic pancreatic cancers (15, 16). Several other CLDN18.2-directed cell- and antibody-based approaches are in development.

In this study, we report the preclinical development and evaluation of an autologous CLDN18.2-directed TAC T-cell therapy (TAC01-CLDN18.2) in models of solid tumors. Our data show that TAC01-CLDN18.2 effectively rejected gastrointestinal and pancreatic tumor xenografts in the absence of notable on-target/off-tumor or off-target effects. Therefore, T cells engineered with the CLDN18.2-TAC have the potential to become an effective approach in treating CLDN18.2-positive solid tumors. A clinical phase I/II study is currently investigating the safety and efficacy of TAC T cells in subjects with unresectable, locally advanced, or metastatic CLDN18.2-positive solid tumors (NCT05862324; ref. 17).

## Materials and Methods

### DNA constructs and vectors

All constructs were codon-optimized and cloned using standard methods. Synthetic DNA fragments encoding reporter genes, CLDN18 proteins, and TAC receptors were purchased from GeneArt, Thermo Fisher Scientific, and Integrated DNA Technology (IDT). CLDN18.2-directed single-domain antibody [variable heavy domain of heavy chain (VHH), nanobody] candidates were obtained from a phage display library derived from peripheral B cells of alpacas (*Vicugna pacos*) immunized with recombinant human CLDN18.2 peptide (Sanyou Biopharmaceuticals). Select nanobodies were further optimized to enhance the specificity, affinity, and degree of humanization, resulting in the final VHH clone NA3SH1-T4-hVH6 (hVH6). Due to the close homology of CLDN18.2 with CLDN18.1, potential cross-reactivity with recombinant CLDN18.1 was ruled out at each stage of hVH6 development through counter-screening against recombinant CLDN18.1. The structure of the final CLDN18.2-TAC receptor consists of an N-terminal CD8α leader peptide, hVH6, c-myc tag, anti-CD3ε scFv, and the transmembrane and cytosolic domains of CD4. An analogous second-generation CAR construct was generated comprising the huIgG κ leader peptide, hVH6, c-myc tag, a hCD8α linker, and the transmembrane and cytosolic domains of CD28 fused to CD3ζ. Lentiviral vectors encoding chimeric receptors were either produced in-house, using the pCDH expression system from System Biosciences and a lentiviral packaging mix from Thermo Fisher Scientific using the Thermo Fisher virus manufacturing system (A35684) and associated protocol (Revision G.0), or obtained from Lentigen Technology. For engineering cancer cell lines, either a custom lentiviral expression vector plasmid was synthesized and used or pCDH vectors were used (custom vector, OriGene Technologies Inc. or System Biosciences). Genes of interest were co-expressed with fluorescence markers such as an enhanced fluorescence version of GFP. GFP used as a cellular marker was derived from published literature (18). The nuclear-localized GFP (nGFP) cell marker protein was designed by fusing GFP to histone H2B type 1-J. DNA sequences are provided in Supplementary Table S1.

### Proteome array study

Soluble his-tagged hVH6-GFP fusion protein was expressed as a secreted protein in mammalian HEK293F cells using a custom lentiviral expression vector system from OriGene Technologies Inc. and purified using standard nickel affinity chromatography (QIAGEN). Specificity testing of the recombinant protein was performed by Integral Molecular using their proprietary membrane proteome array screen comprising >6,000 human membrane proteins (19, 20). Briefly, binding data of hVH6-GFP done in replicates across the protein library, including controls, were established via a fluorescently labeled secondary antibody, transformed, and plotted as a unitless, ranked, nonlinear score for target binding. Test ligand interactions with any target identified by membrane proteome array screening were confirmed in a second flow cytometry experiment using serial dilutions of the test antibody, and the target identity was confirmed by sequencing.

### T-cell manufacturing

CLDN18.2-TAC T cells were manufactured using purified CD4/CD8 T cells from healthy donors (New York Blood Center or HemaCare) during a 9-day process in either the G-Rex system from Wilson Wolf or the Cocoon platform from Lonza. TAC T cells derived from the latter process utilizing the CLDN18.2-TAC vector from Lentigen are referred to as TAC01-CLDN18.2. As negative controls, nontransduced (NTD) T cells from the corresponding donors were generated following the same manufacturing processes without *Lentivirus*. TAC-positivity was assessed via anti-myc flow cytometry.

### Cell culture

Human cancer cells were obtained from the ATCC, the European Collection of Authenticated Cell Cultures, the Deutsche Sammlung von Mikroorganismen und Zellkulturen GmbH, and AcceGen Biotechnology. Primary cells representing normal human tissues were purchased from Lonza, Promocell, and Cell Applications. All cells were cultured according to the manufacturers’ instructions and listed in Supplementary Table S2. Where indicated, cancer cells were engineered with reporter or CLDN18 genes using lentiviral approaches described above. Briefly, cells were incubated with lentiviral particles at multiplicity of infection(s) ranging from 1 to 20 in a 24-well dish, and cultures were expanded for one to two passages before being subjected to antibiotic selection.

For functional assays, T cells were thawed, rested overnight, and counted using the Vi-CELL BLU instrument (Beckman Coulter). Target cells were seeded 1 day prior to adding T cells for coculture experiments. T-cell activation was evaluated after a 4-hour coculture in the absence or presence of GolgiPlug (BD Bioscience) via flow cytometric assessment of cell surface CD69 and intracellular cytokine levels, respectively. For measuring T-cell proliferation, target cells were reseeded in media containing 50 μg/mL mitomycin-C (Enzo) at 37°C for 45 minutes, followed by three washes in PBS and centrifugation. In parallel, T cells were stained with CellTraceViolet (Sigma-Aldrich) for 20 minutes at 37°C and washed in PBS. Then, T cells were cocultured with target cells for 4 days and quantified by flow cytometry. To assess cytotoxicity, target cells expressing nGFP were combined with T cells at varying effector-to-target (E:T) ratios in media containing 1 μmol/L DRAQ7 cell death dye (Thermo Fisher Scientific) and imaged every 8 hours for a total of 5 days using Cytation 5 Cell Imaging Reader (Agilent). The area of live cells was calculated by subtracting the nGFP/DRAQ7 co-stain from the total nGFP and plotted against time. The AUC value of each killing curve was calculated and presented relative to AUC values obtained from target cells alone (100%). Recursive killing was determined by coculturing T cells and target cells in an E:T = 3:1 ratio over repeated rounds of 3 or 4 days until T cells became inactive and/or experienced cell death. nGFP levels were measured via live-cell imaging at the beginning and end of each round, after which T cells were harvested, counted, and used in a subsequent round with fresh target cells. Tumor spheroid assays, IHC, and the corresponding data analysis were performed by CRO InSphero (Schlieren) using T cells provided by Triumvira. Briefly, spheroids were formed by culturing N87^CLDN18.2^ or N87 cells together with human cancer-associated fibroblasts (CAF; InSphero). Spheroid growth was monitored using brightfield and GFP images, which were taken using a Leica fluorescence microscope (days 0–7). Following spheroid formation, either TAC or nonengineered T cells were added at ratios 2, 5, and 20:1 and cocultured for 7 days. For the duration of the coculture, the morphology of 3D InSight tumor microtissues was monitored in the AkuraTM384-well plate by performing a fluorescent microscopy analysis. Images of one representative microtissue per experimental condition were obtained using GFP and brightfield channels on the Leica Dmi8 microscope with a 10× magnification lens. Merged images were then generated using the ImageJ software. The fluorescence signals from GFP-labeled tumor cells within the 3D tumor microtissues were measured on a Spark 10M multimode microplate reader. Relative fluorescence units were recorded, background corrected, and plotted in GraphPad Prism. For IHC stainings, microtissues were harvested (*n* = 24 microtissues per sample) and fixed with 4% paraformaldehyde. The fixed samples were embedded in agarose for further paraffin embedding, sectioning, and staining. Antibodies used for IHC analysis are listed in Supplementary Table S3. All images were taken using a Leica DMi8 microscope with a DMC4500 camera with a 40× objective lens.

### Flow cytometry

Flow cytometry analysis was done using the ID7000 spectral flow cytometer (Sony Biotechnology) in a 3-color configuration (488, 637, and 405 nm). Antibodies used were properly titrated before use at appropriate concentrations and are listed in Supplementary Table S3. Staining of all samples was done at 4°C. Data were analyzed using FCS Express (version 7.12.0007). Gating strategies and representative dot plots are shown in Supplementary Materials–Flow Cytometry.

### *In vivo* methods

All animal work was carried out in compliance with federal laws for animal ethics (Institutional Animal Care and Use Committee/Canadian Council On Animal Care) at TD2 and the vivarium at McMaster University. Mouse strains used were either NOD/SCID gamma (NSG; NOD.Cg-*Prkdc*^*scid*^*Il2rg*^*tm1Wjl*^/SzJ) or NSG-MHC I/II DKO (NOD.Cg-*Prkdc*^*scid*^*H2-K1*^*b-tm1Bpe*^*H2-Ab1*^*g7-em1Mvw*^*H2-D1*^*b-tm1Bpe*^*Il2rg*^*tm1Wjl*^/SzJ) from The Jackson Laboratory. Tumor cells were inoculated via s.c. injections at the hind flank and grown into established solid tumors (50–100 mm^3^) before T cells were administered via tail-vein injection as a single bolus. Secondary tumors as part of tumor rechallenge studies were implanted onto the opposite flank. Tumors were monitored biweekly using calipers. Blood was taken via submandibular bleeds, and tissues were collected in either 10% formalin for histologic work or flash-frozen for genomic DNA (gDNA) extraction. Histology was assessed at Nationwide Histology via hematoxylin and eosin staining and IHC analysis using a pan-specific Claudin 18 antibody (Thermo Fisher Scientific, clone 34H14L15, cat. # 700178), which was visualized using STAT-Q Monovalent-Rabbit Linking antibody (NB314KR-60). Blinded histologic analysis of tissue samples was performed by Dr. Ian Welch (Welch Veterinary Forensic and Pathology Services). During all studies, animals were monitored for body weight and other clinical observations related to animal activity and behavior, posture, skin integrity, coat, and quality of feces to evaluate potential toxicities, including GvHD. Serum cytokine analysis was performed by Eve Technologies using their Human Cytokine Proinflammatory Focused 15-Plex bead-based Discovery Assay Array.

### Droplet digital PCR assay

Mouse tissues were homogenized (*n* = 6) using Miltenyi gentleMACS Octo Dissociator (Miltenyi Biotec). RNA and gDNA were isolated using QIAGEN kits (RNeasy Plus Mini, DNeasy Blood and Tissue, or QIAamp DNA Micro) according to the manufacturer’s instructions (QIAGEN). RNA was converted to cDNA using iScript cDNA Synthesis Kit (Bio-Rad and amplified in the C1000 Touch thermocycler (Bio-Rad). Primers specific to CLDN18.2, CLDN18, and huUCHT1 were custom ordered from IDT and Bio-Rad and are shown for the forward, reverse, and detection probes, respectively, in the 5′→3′ orientation as follows. UCHT1: CCAGTCAGGACATCCGTAAT, AGGCGGGAGGTATAGTAAAT, and ACAGAAACCAGGAAAAGCTCCGA; CLDN18.2: AGAGAGCTCTGGCTTCACCGAG, AATGGCACCCAGGACGATGC, and CGGGGCTACTTCACCCTGCTGG; CLDN18.2 (codon-optimized): GCCGCCACCTGTATGGATCA, AGTTGAACACGGCGGTCACA, and TCTACCCAGGACCTGTACAACAACCC; and RPP30: ATTACGCTGTGTGTGGATTT, AGTCCTCTATAAGGGAATGAGA, and TGCCTCTTCTGCCCTGTTCTGT. Both gDNA and cDNA were used in the QX200 instrument (Bio-Rad Laboratories) at 100 ng/reaction in a droplet digital PCR assay (ddPCR). ddPCR was performed following standard procedures as per Bio-Rad Droplet Digital PCR Application Guide (Bulletin 6407). The reagents used were nuclease-free water (IDT Technologies, 11-05-01-04), ddPCR supermix for probes (no dUTP), 500 reactions (Bio-Rad, 1863024), DG8 cartridges for the QX100/QX 200 Droplet generator (Bio-Rad, 1864008), Droplet generation oil for probes (Bio-Rad, 1863005), ddPCR 96-well semi-skirted plates (Bio-Rad, 12001925), Droplet generator DG8 gasket (Bio-Rad, 1863009), PCR plate heat seal, and foil, pierceable (Bio-Rad, 1814040). The *TAC*-specific ddPCR assay (huUCHT1) was qualified and exhibited a lower limit of detection and a lower limit of quantitation (LLOQ) of 60 and 330 copies per μg gDNA, respectively.

### Data analysis and statistical methods

All flow data were analyzed using the FCS Express software (*De Novo* Software). Cytation images were captured and analyzed using the Gen5 software (Agilent). ddPCR data analysis was performed using the QX Manager Software (Bio-Rad). All numerical data were analyzed using Microsoft Excel and GraphPad Prism software. Statistical significance was determined via one- and two-way ANOVA algorithms provided by GraphPad.

### Data availability

All data associated with this study are presented in the paper or Supplementary Materials. Raw data generated in this study are available upon reasonable request from the corresponding authors.

## Results

### Selection of the CLDN18.2 binder and the CLDN18.2-TAC construct

**Figure 1.**
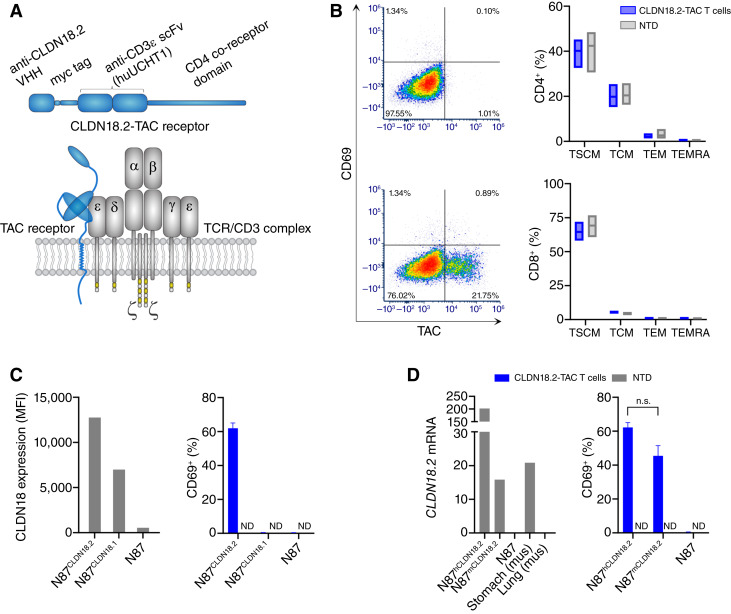
TAC01-CLDN18.2 phenotype and specificity. **A,** Schematic of the CLDN18.2-TAC receptor (blue). **B,** Left, Flow cytometry analysis of surface CD69 (*y*-axis) vs. TAC receptor (*x*-axis) of NTD control (top) and CLDN18.2-TAC T cells (bottom). Right, Histograms of flow cytometry data from three TAC01-CLDN18.2 products and three corresponding NTD products showing the minimum, maximum, and mean of CD4^+^ (top) and CD8^+^ (bottom) T-cell memory phenotypes gated as follows: TSCM (CD45RA^+^, CCR7^+^, CD62L^+^), TCM (CD45RA^−^, CCR7^+^, CD62L^+^), TEM (CD45RA^−^, CCR7^−^, CD62L^−^), and TEMRA (CD45RA^+^, CCR7^−^, CD62L^−^). **C,** Lack of cross-reactivity against human CLDN18.1. Left, Flow cytometry analysis of CLDN18.1 and CLDN18.2 surface expression in N87 cells ectopically expressing human CLDN18.1 and CLDN18.2 using a pan-specific anti-CLDN18 antibody recognizing both isoforms. Right, Flow analysis of CD69 surface expression in CLDN18.2-TAC T and NTD control T cells after a 4-hour coculture with target cells, as indicated. **D,** Cross-reactivity with murine CLDN18.2. Left: ddPCR mRNA analysis of human and mouse *CLDN18.2* in N87 cells ectopically expressing the human (hCLDN18.2) and murine (mCLDN18.2) homologs as well as stomach and lung tissues from naïve NSG mice. Right, Flow analysis of CD69 surface expression in CLDN18.2-TAC T and NTD cells after a 4-hour coculture with target cells. Averages and SDs derived from three cell products manufactured from three separate donors are shown (**C** and **D**). MFI, median fluorescence intensity; mus, Mus musculus v. alba; ND, not detected; n.s., not statistically significant (two-way ANOVA, alpha = 0.05); TEM; effector memory T cells; TEMRA; T cells that re-express CD45RA; TCM; central memory T cells; TSCM, stem cell-like memory T cells.

The development of the CLDN18.2-TAC receptor involved the screening of multiple TAC constructs comprising different CLDN18.2 nanobodies and various TAC scaffolds in a series of functional *in vitro* and *in vivo* models. Preferred TAC receptors were those that were robustly expressed on the surface of T cells combined with (i) a lack of autoactivation or tonic signaling and (ii) potent and long-lasting T cell–induced antitumor activity associated with low cytokine secretion, all of which are hallmarks of the TAC technology (1). The final CLDN18.2-TAC construct comprises an N-terminal CD8α leader peptide followed by the hVH6 CLDN18.2–directed humanized camelid nanobody and the TAC receptor scaffold. The latter consists of a humanized UCHT1-based CD3ε scFv (huUCHT1) as well as the transmembrane and cytosolic domains of the human CD4 co-receptor (Fig. 1A). An extracellular human c-myc tag positioned between the CLDN18.2 and huUCHT1 binder sequences enabled the detection of TAC-positive T cells by flow cytometry using an anti-myc antibody. An analogous CLDN18.2-CAR construct was generated encoding an N-terminal CD8α leader peptide followed by the hVH6 CLDN18.2 binder, c-myc tag, CD28 transmembrane and cytoplasmic domains, and cytoplasmic portion of CD3ζ. CLDN18.2-TAC T cells were generated via lentiviral transduction and—when produced using a GMP-compliant lentiviral vector (Lentigen) and the Cocoon manufacturing technology—are referred to as TAC01-CLDN18.2 (21). The donor-derived T-cell product is generally characterized by 20% to 60% TAC positivity [Fig. 1B (left)], a lack of tonic signaling as noted by a lack of CD69 surface expression in the absence of antigen [Fig. 1B (left)], and a majority of T cells carrying stem cell-like memory T cells (TSCM; CD45RA^+^, CCR7^+^, and CD62L^+^) and central memory T cell (TCM; CD45RA^−^, CCR7^+^, and CD62L^+^) memory markers [Fig. 1B (right)].

### Specificity and cross-reactivity of the CLDN18.2-TAC

The specificity of the hVH6 nanobody was evaluated in a membrane proteome array study using recombinant hVH6-GFP protein produced in HEK293F cells. The protein array consisted of >6,000 human membrane proteins that represent ∼94% of all single-pass, multi-pass, and glycosylphosphatidylinositol-anchored human proteins and contained all CLDN family members with known sequences (24 of 27 members), including CLDN18.1. The results supported that hVH6-GFP binding is exclusive to CLDN18.2 and does not occur with other human membrane proteins (Supplementary Fig. S1).

Next, we evaluated the specificity of the full-length CLDN18.2-TAC receptor expressed on the surface of T cells. To this end, the reactivity of TAC01-CLDN18.2 was tested in coculture with CLDN18.2-negative N87 gastric cancer cells and N87 cells engineered to overexpress either human CLDN18.1 or CLDN18.2 (N87^CLDN18.1^ or N87^CLDN18.2^, respectively). CLDN18.2 and CLDN18.1 expression on engineered cells was confirmed by flow cytometry via a CLDN18-reactive antibody that recognizes both isoforms. T-cell activation was determined by surface CD69 expression, a sensitive and early indicator of T-cell activation (22). The results show that TAC01-CLDN18.2 was solely activated when cocultured with CLDN18.2-expressing N87^CLDN18.2^ cells, suggesting that TAC01-CLDN18.2 does not cross-react with CLDN18.1 (Fig. 1C).

Previous studies demonstrated the high degree of conservation of the CLDN18.2 sequence, functional domains, and expression patterns in various tissues across human, mouse, and other mammalian species and that orthologous CLDN18.2 mRNA transcripts and proteins were exclusive to differentiated gastric cells in these species (23). Because the amino acid sequence of the targeted CLDN18.2 epitope is identical between a mouse and a human, we also evaluated the potential for cross-reactivity of TAC01-CLDN18.2 with murine CLDN18.2. TAC01-CLDN18.2 was cocultured with N87 cells engineered to express either human CLDN18.2 (hCLDN18.2) or murine CLDN18.2 (mCLDN18.2) and interrogated for CD69 surface expression after 4 hours. CD69 expression was absent in T cells stimulated with CLDN18.2-negative cells (Fig. 1D). In contrast, CD69 was elevated to similar levels in both N87^mCLDN18.2^ and N87^hCLDN18.2^ cocultures, indicating strong cross-reactivity of TAC01-CLDN18.2 with cells expressing murine CLDN18.2. Thus, in accord with previously published reports, our data confirm the expression of CLDN18.2 in murine gastric tissue (Fig. 1D) and support the use of NSG mice for evaluating potential on-target/off-tumor effects of CLDN18.2-TAC T cells *in vivo*.

### Activation of TAC T cells by cancer cells endogenously expressing CLDN18.2

**Figure 2.**
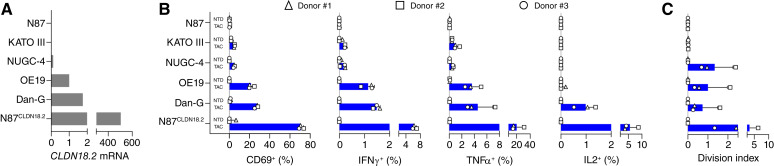
CLDN18.2-specific activation of TAC01-CLDN18.2. **A**, CLDN18.2 ddPCR mRNA analysis of cancer cells naturally and ectopically expressing CLDN18.2. Expression is shown as CLDN18.2 mRNA copy number relative to the reference gene *RPP30* and normalized to OE19 cells. **B** and **C,** Flow cytometry analysis of surface CD69 and intracellular cytokine expression in CLDN18.2-TAC and NTD T cells after a 4-hour coculture (**B**) and T-cell proliferation after 4-day coculture (**C**) with target cells described in (**A**). All data are shown as averages with SDs derived from experiments done with three cell products, each from a separate donor, as indicated by the different symbols. Whereas the level of TAC01-CLDN18.2 activation against CLDN18.2-expressing target cells varied across the various assays and target cells, overall activation was significantly higher in TAC01-CLDN18.2 compared with NTD controls (see statistical analysis in Supplementary Table S4).

The activity of TAC01-CLDN18.2 was further evaluated in a series of *in vitro* cocultures with CLDN18.2-positive and CLDN18.2-negative human solid tumor cells. We confirmed varying CLDN18.2 expression in multiple cell lines known to endogenously express CLDN18.2, including KATO III (gastric carcinoma), NUGC-4 (gastric signet ring cell carcinoma), OE19 (gastroesophageal carcinoma), and Dan-G (pancreatic carcinoma; refs. 24–27). N87 and N87^CLDN18.2^ cells were used as negative and positive controls, respectively (Fig. 2A). CLDN18.2-TAC T cells produced from three different donors were cocultured with target cells for 4 hours to evaluate CD69, TNFα, IFNγ, and IL2 expression (Fig. 2B; Supplementary Table S4) and for 4 days to evaluate T-cell proliferation (Fig. 2C; Supplementary Table S4). The corresponding NTD T cells for each donor served as additional negative controls. The results show antigen-specific CD69 expression on TAC01-CLDN18.2 cells after incubation with CLDN18.2-positive cancer cells. CD69 expression was most elevated after stimulation with N87^CLDN18.2^ cells, followed by Dan-G and OE19, and least in the NUGC-4 and KATO III cocultures, potentially indicating a positive association with CLDN18.2 expression on target cells. Similarly, TAC T cell–mediated cytokine increases in T cells were specific to CLDN18.2-positive target cells and were notably greater when exposed to target cells with higher CLDN18.2 expression. T-cell proliferation was also exclusive to stimulation with CLDN18.2-positive cancer cells. However, unlike CD69 and cytokine data measured at 4 hours, no antigen-dependent increase in T-cell proliferation was observed on day 4. Furthermore, KATO III which had the lowest levels of *CLDN18.2* mRNA, did not induce any TAC01-CLDN18.2 proliferation, suggesting that the low CLDN18.2 levels in KATO III cells may have been sufficient to induce activation markers on a small percentage of CLDN18.2-TAC T cells (<5.2%) but insufficient to stimulate T-cell proliferation. None of the NTD controls induced significant CD69, cytokine expression, or T-cell proliferation, further indicating that the observed T-cell activities were CLDN18.2 antigen–dependent.

### Potency of CLDN18.2-TAC T cells against cultured cancer cells

**Figure 3.**
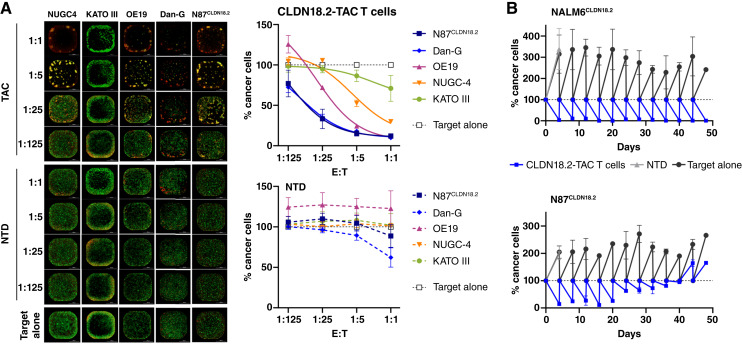
Cytotoxicity of CLDN18.2-TAC T cells against gastric, gastroesophageal, and pancreatic cancer cells. **A,** TAC01-CLDN18.2 potency in a 5-day coculture. Tumor cells were cocultured with TAC01-CLDN18.2 at different E:T ratios and monitored via nGFP during live-cell imaging for 120 hours. Left, Representative images taken at the end of the 120-hour cocultures. Green: nGFP-engineered tumor cells; red: DRAQ7 death dye. Right, Cytotoxicity per E:T ratio as measured by AUC values of killing curves normalized to target alone. A statistical analysis is provided in Supplementary Table S5. **B,** Recursive killing assay. CLDN18.2-TAC T cells were cocultured with GFP-expressing NALM6^CLDN18.2^ or N87^CLDN18.2^ target cells at a 3:1 E:T ratio in rounds of 4 days over the course of 12 total rounds and 48 days. At the end of each round, cytotoxicity was evaluated by measuring GFP fluorescence emitted from the cancer cells and expressed as percentage of cancer cells relative to cancer cells at the beginning of each round (100%). Then, T cells were carried forward into a new round with fresh tumor cells at a 3:1 ratio. As controls, tumor cells alone and tumor cells cultured with NTD cells were used. The NTD coculture did not progress beyond round 1 as all T cells died in the absence of the TAC receptor. Averages and SDs from three replicates are shown, except for rounds 11 and 12 (NALM6^CLDN18.2^) and round 12 (N87^CLDN18.2^), for which there were duplicates. All data points reached statistical significance (two-way ANOVA, alpha = 0.05, with Sidak’s post test).

TAC01-CLDN18.2–induced cytotoxicity was analyzed using multiple assays. First, CLDN18.2-positive target cells, which constitutively expressed nGFP to allow for monitoring of tumor cells via live-cell imaging, were cocultured with TAC T cells for 5 days at E:T ratios ranging from 1:125 to 1:1. Because the nGFP protein can remain intact for an extended period after target cell lysis, we separately monitored cell death using the DRAQ7 dye. Individual killing curves were established by quantifying the nGFP fluorescence signal that colocalized with the cell death dye (Supplementary Fig. S2), and cytotoxicity was determined by plotting AUC values of each curve per E:T ratio against time relative to the AUC value derived from target cells alone. As shown in Fig. 3A, all TAC01-CLDN18.2 products derived from three different donors elicited dose-dependent tumor cell killing in all cancer cocultures which, in some cocultures, was already observed at the lower E:T ratios (1:125–1:25). The level of cytotoxicity appeared to be associated with increasing concentrations of CLDN18.2 expression on target cells, such that greatest activity was observed in N87^CLDN18.2^ and Dan-G cocultures and the lowest activity was observed in KATO III cocultures (Fig. 3A, Supplementary Table S5). In contrast, NTD T cells failed to exhibit any antitumor activity.

Next, we assessed the anti-tumor activity of CLDN18.2-TAC T cells in a recursive killing assay in which TAC T cells were repeatedly exposed to fresh batches of cancer cells over multiple rounds of 3- to 4-day cocultures until the TAC T culture becomes inactive and/or experiences cell death. Thus, this assay is designed to mimic a scenario of chronic antigen exposure, thereby stress-testing the functional persistence of TAC T cells. The results demonstrated that CLDN18.2-TAC T cells were able to eradicate and control CLDN18.2-expressing NALM6^CLDN18.2/GFP^ acute lymphoblastic lymphoma cells and N87^CLDN18.2/nGFP^ cells for up to 48 days over 12 consecutive rounds of repeated cocultures (Fig. 3B). In a head-to-head comparison against CLDN18.2-directed second-generation CAR T cells derived from the same two donors and hVH6 antigen-binding domain, CLDN18.2-TAC T cells exhibited greater expansion with a notably higher proportion of CD8^+^ T cells, lower levels and delayed onset of T-cell exhaustion, and overall greater and longer-lasting cytotoxicity (Supplementary Fig. S3). Thus, these results support the rationale of the TAC design as observed previously in another TAC versus CAR head-to-head comparison in a HER2-positive solid tumor model and that TAC can enhance the functional persistence of T cells (1, 3, 28).

**Figure 4.**
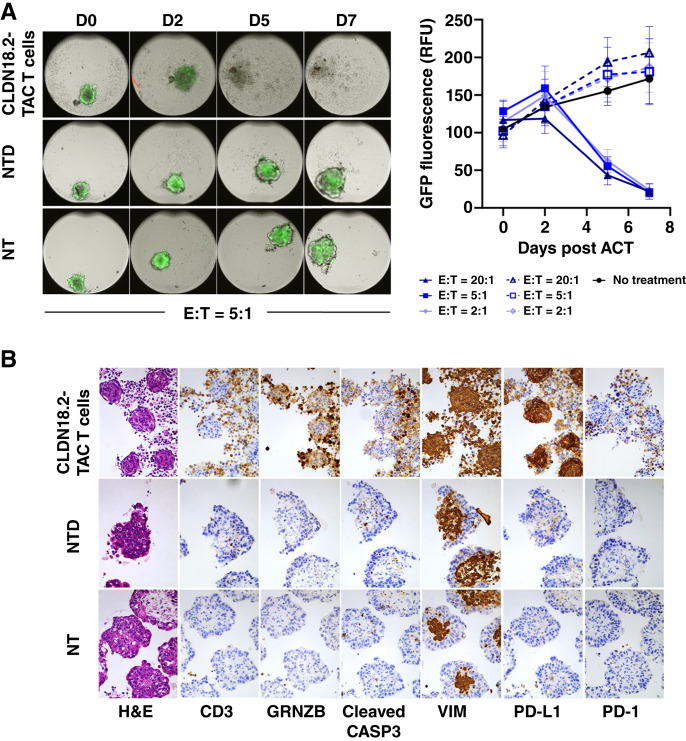
Clearance of gastric tumor spheroids by CLDN18.2-TAC T cells. **A,** Cytotoxicity of TAC01-CLDN18.2 against N87^CLDN18.2^ tumor spheroids. GFP-expressing N87^CLDN18.2^ spheroids aggregated with CAFs were cocultured with CLDN18.2-TAC T cells at various E:T ratios for 7 days. Left: Microscopy images of tumor spheroids (E:T = 5:1). Right: Quantitation of GFP fluorescence over time. Dotted lines denote NTD cocultures. Averages and SDs of experiments done in triplicates are shown. **B,** IHC analysis of spheroids cultured at E:T = 5:1 and collected on day 2. H&E, hematoxylin and eosin; NT, not treated; RFU, relative fluorescence units; VIM, vimentin.

Finally, we tested the cytotoxic potential of TAC01-CLDN18.2 against gastric tumor spheroids formed by N87^CLDN18.2/GFP^ expressing GFP. Cancer cells were mixed with CAFs to achieve optimal 3D growth and spheroid formation. TAC T cells were added at different E:T ratios and incubated for 7 days. Cytotoxicity was determined by measuring fluorescence levels in the remaining tumor spheroids. The data showed potent killing of N87^CLDN18.2^ tumor spheroids by TAC01-CLDN18.2 (Fig. 4). Cytotoxicity was observed at all E:T ratios, resulting in near-complete spheroid eradication. IHC analysis of spheroids collected during the assay revealed effective T-cell infiltration into the 3D tumor structure (CD3), signs of T-cell activation (granzyme B, PD-1), and apoptotic tumor cells (cleaved caspase 3) while sparing CAFs (vimentin; Fig. 4B). Many of the cells in the TAC01-CLDN18.2 cocultures also expressed PD-L1, an IFNγ-inducible surface marker that is likely a result of IFNγ secretion by activated TAC T cells (29). In contrast, N87^CLDN18.2^ spheroids cultured alone or with NTD cells remained unaffected, continued to grow, and did not upregulate PD-L1. In summary, *in vitro* coculture experiments demonstrate that TAC01-CLDN18.2 activation and cytotoxicity are specific to cancer cells expressing the CLDN18.2 antigen and are induced by multiple CLDN18.2-expressing cancer cells of gastric, gastroesophageal, and pancreatic origin. The data also suggest a potential correlation between TAC01-CLDN18.2 activity/cytotoxicity and CLDN18.2 mRNA expression levels on target cells, suggesting greater TAC T-cell activity in the presence of greater CLDN18.2 antigen densities on tumor cells.

### *In vivo* pharmacology of CLDN18.2-TAC T cells

**Figure 5.**
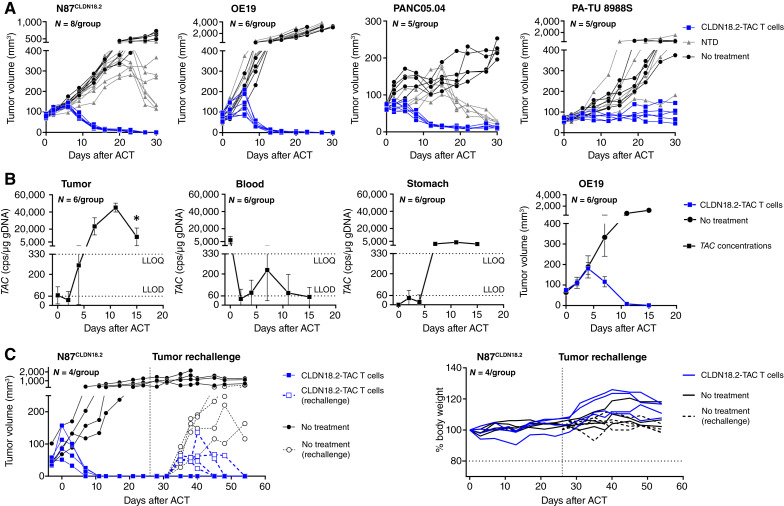
Pharmacology of CLDN18.2-TAC T cells in various CLDN18.2-positive tumor mouse models. **A, ***In vivo* efficacy of CLDN18.2-TAC T cells against s.c. solid tumors. Tumor volumes of individual animals are shown (*n* = 5–8/grp). Animals were administered a single i.v. dose of 6 × 10^6^ TAC01-CLDN18.2 or NTD control cells. **B,** Pharmacokinetics of TAC01-CLDN18.2. NSG mice carrying established s.c. OE19 tumor xenografts were treated with a single i.v. dose of 6 × 10^6^ TAC01-CLDN18.2. Tumor, blood, and stomach samples were collected at various time points, processed to gDNA, and evaluated by *TAC*-specific ddPCR. Averages and SDs derived from six animals per time point are shown. *, Averages and SDs were derived from only two tumor samples due to tumor clearance in four of six mice. Right, Average tumor volume of mice in the pharmacokinetic study. **C,** CLDN18.2-TAC T cell efficacy during tumor rechallenge. NSG/MHC-DKO mice engrafted with N87^CLDN18.2^ tumor xenografts were treated once with 6 × 10^6^ CLDN18.2-TAC T cells (day 0). Two weeks after the primary tumors regressed, mice were rechallenged with N87^CLDN18.2^ tumor cells given in the opposing hind flank (day 26). Separate groups of mice receiving no treatment were used as negative controls during the first and second tumor challenges. Left, Tumor volumes of individual animals. Right, Mouse body weights relative to day 0. A statistical analysis of efficacy data is shown in Supplementary Table S6. NT, not treated.

To establish efficacy across multiple cancer indications, TAC01-CLDN18.2 products were tested in mice carrying established, s.c. CLDN18.2-positive tumor xenografts, including N87^CLDN18.2^ and OE19, both previously used *in vitro*, and PANC 05.04 and PA-TU 8988S pancreatic carcinoma xenografts (30, 31). In all studies, TAC01-CLDN18.2 was administered as a single i.v. tail-vein injection at a dose of 6 × 10^6^ TAC+ T cells per mouse. Tumors in three of the mouse models (OE19, N87^CLDN18.2^, and PANC 05.04) showed rapid regression upon TAC01-CLDN18.2 treatment, leading to complete tumor clearance that was achieved 24 days after adoptive cell transfer (ACT) or earlier (Fig. 5A; Supplementary Table S6). TAC01-CLDN18.2 also strongly inhibited PA-TU 8988S tumors, such that there was minimal tumor growth after treatment (tumor growth inhibition = 83% vs. not treated, day 30). In contrast, tumors in untreated animals, or those treated with an equivalent dose of NTD cells continued to grow. At much later time points, TAC-unrelated tumor regression by NTD cells was observed in mice carrying PANC 05.04 on day 19 and N87^CLDN18.2^ on day 27 but not OE19 and PA-TU 8988S xenografts. The TAC01-CLDN18.2–induced efficacy was reproducible across different donor-derived products (Supplementary Fig. S4) and dose-dependent, with maximal tumor clearance achieved at the 6 × 10^6^ dose level and lower degrees of tumor regression at the 3 × 10^6^ and 1 × 10^6^ dose levels (Supplementary Fig. S5).

Then, we established the *in vivo* TAC T-cell expansion profile during and after the regression of OE19 tumors. Because TAC T cells were able to eliminate tumors on or before day 15 post-ACT, tissues were collected at 30 minutes as well as at 2, 4, 7, 11, and 15 days after treatment. The final biodistribution data set comprised of data from three female and three male mice per sampling time point collected from tumor, blood, and a series of normal tissues, including bone marrow, spleen, lung, liver, heart, stomach, esophagus, skin, pancreas, kidney, colon, intestine, brain, and gonads. Tissues collected from naïve or nontreated tumor-bearing mice (day 15 post-ACT) were used as negative controls. All pharmacokinetic assessments were done using tissue-derived genomic DNA in a qualified ddPCR assay targeting a sequence within the *TAC* transgene not shared with the mouse and human genomes. The data showed transiently detectable *TAC* concentrations in blood 30 minutes after TAC01-CLDN18.2 administration which decreased by day 2 below the lower limit of detection of the ddPCR assay (Fig. 5B). On day 4 and subsequent days, TAC concentrations in blood increased again but remained generally at low levels with averages below the LLOQ until the end of the study (day 15). The recurrence of *TAC* levels in blood coincided with substantially increasing tumor concentrations which were first notable on day 7, with tumor concentrations 104-times higher than those in blood, and reached peak levels on day 11 that were 585-times higher in tumor than those observed in blood. On day 15, tumor *TAC* levels decreased but remained 199-times higher than those in blood. Interestingly, *TAC* levels were generally low in normal tissues, typically below or close to the LLOQ of the ddPCR assay (Supplementary Fig. S6; Supplementary Table S7). At the 30-minute time point after TAC01-CLDN18.2 administration, *TAC* levels in all normal tissues but blood, lung, and liver were either not detectable or below the LLOQ of the ddPCR assay. At later time points, particularly during TAC T-cell expansion in the tumor (days 7–15), *TAC* concentrations were low in lung, liver, spleen, stomach, and esophagus and not quantifiable in the pancreas, heart, colon, intestine, and brain. Interestingly, normal stomach tissues never presented *TAC* levels greater than those observed in other CLDN18.2-negative tissues nor did they increase at later time points, suggesting a lack of antigen-driven TAC T-cell accumulation or proliferation in normal stomach. Taken together, the kinetics of *TAC* concentrations in the tumor and normal tissues indicate antigen-specific trafficking and accumulation/proliferation of CLDN18.2-TAC T cells to and at its intended target tumor site and corresponds with the kinetics of tumor regression.

Data obtained with the *in vitro* repeat killing assay suggest that CLDN18.2-TAC T cells can persist and remain functional in an environment involving chronic antigen-exposure. To determine the durability of TAC T-cell responses *in vivo*, we evaluated the functional persistence of CLDN18.2-TAC T cells in a 54-day tumor rechallenge experiment. Because GvHD can occur within 30 days of human T-cell engraftment and confound antigen-specific T-cell responses, these studies were conducted using MHC double-knockout mice which are less susceptible to GvHD (32). As shown in Fig. 5C, infusion of CLDN18.2-TAC T cells led to complete regression of the initial N87^CLDN18.2^ tumor xenograft. Fourteen days after tumor clearance (day 26 post-ACT), mice were rechallenged with fresh CLDN18.2-expressing tumor cells injected into the opposite flank. The tumorigenicity of the fresh tumor implant was confirmed in a separate group of untreated animals, which grew into big tumors as expected. In TAC T cell–treated animals, however, the newly grafted tumor cells developed into only small tumors, were all controlled, and, eventually, were completely rejected. Because no additional CLDN18.2-TAC T cells were administered, these results suggest that the second tumor inoculation was rejected by the presence of persisting TAC T cells. As previously observed in this tumor model, two mice treated with NTD showed varying degrees of TAC-unrelated antitumor responses which, however, was easily distinguishable from the observed CLDN18.2-TAC–specific antitumor response (Supplementary Fig. S7).

Throughout all *in vivo* studies, mice were routinely monitored for potential T cell–induced toxicities, such as GvHD, hyperactivation of T cells, or on-target/off-tumor effects. All animals were monitored for clinical observations and body weights. A select group of animals monitored for up to 91 days after treatment was used to assess serum cytokines on days 3, 7, and 15 after treatment and to examine up to 12 tissues histopathologically by a certified veterinary pathologist in a blinded fashion. The long-term study was done in the MHC DKO mouse model to avoid potential GvH activity and evaluated the effects of a much higher dose to reveal potential toxicities (12 × 10^6^ TAC T). Accordingly, none of the mice developed signs of GvHD, and body weight assessments suggest that TAC T treatment was well tolerated (Fig. 5C; Supplementary Fig. S8). Serum cytokines were elevated only transiently on days 7 to 15, nearing baseline levels by day 15, which aligns with the kinetics of tumor regression and the presence of TAC T cells in this model (Supplementary Fig. 9; Supplementary Table S8). Similarly, the histopathologic analysis of stomach tissues collected on day 91 after treatment failed to detect signs of GvHD or on-target/off-tumor effects in CLDN18.2+ gastric tissue (Supplementary Fig. S10). These results are encouraging and support the thesis around CLDN18.2 as a tumor-selective target. However, given the physiologic differences between tumors in humans and mice, clinical monitoring of potential on-target/off-tumor effects in human subjects is recommended.

### Lack of CLDN18.2-TAC T-cell activity against normal human cells

**Figure 6.**
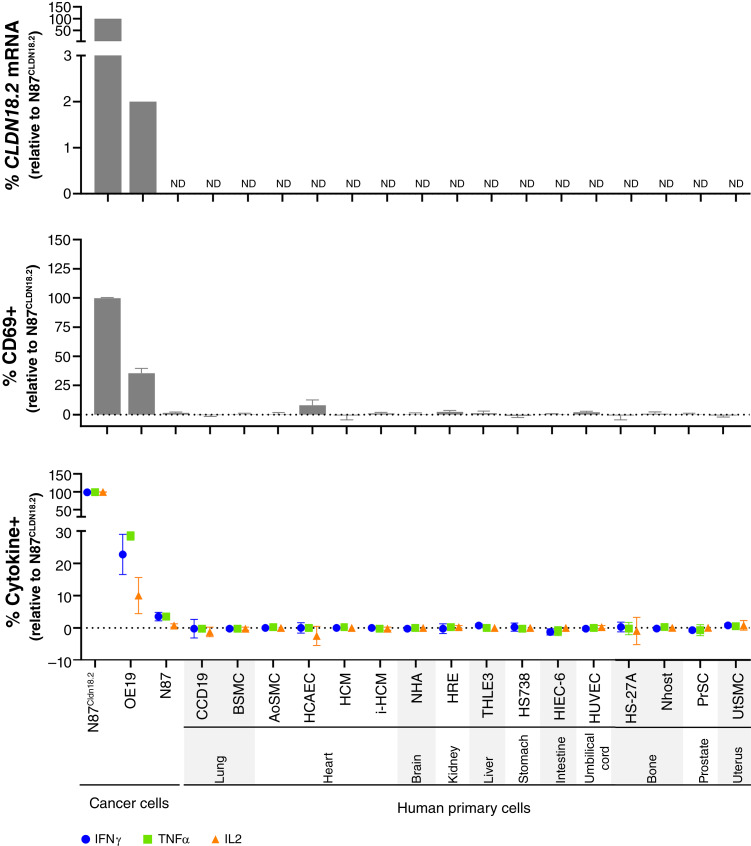
TAC01-CLDN18.2 lacks activity against primary cells representing CLDN18.2-negative normal human tissues. Top, CLDN18.2 mRNA analysis by ddPCR in cancer and human primary cells, as indicated. Data are presented as percent relative to levels in N87^CLDN18.2^ cells (100%). Center/bottom, Flow cytometry analysis measuring proportions of T cells expressing surface CD69 (center) and intracellular cytokines (bottom) determined after a 4-hour coculture of CLDN18.2-TAC T cells with target cells. Data are normalized to data obtained from NTD T cells cocultures to correct for background activity and are plotted relative to values in N87^CLDN18.2^ cells (100%). Data are shown as averages with SDs derived from experiments done with four TAC01-CLDN18.2 products manufactured from three separate donors. CD69 and all cytokines were significantly elevated in T cells cocultured with OE19 target cells compared with the N87 negative controls or all other human cell models. There was no significant difference between the N87 negative control and any of the other human cell models (statistical data are shown in Supplementary Table S9). ND, not detected.

Our previous studies demonstrated that the hVH6 binder exclusively recognized CLDN18.2. To further evaluate the potential of off-target effects, we expanded this analysis and tested whether TAC01-CLDN18.2 reacts to human nonmalignant cells representing vital organs. CLDN18.2-TAC T cells were cocultured with cells representing normal lung, heart, brain, kidney, liver, stomach, intestine, endothelium, bone, prostate, and uterus and assessed for increases in CD69 and cytokine expression thereafter. A ddPCR analysis confirmed that all these cells, including HS738 gastric fibroblasts, lacked endogenous CLDN18.2 expression, in agreement with published reports showing that CLDN18.2 is confined to gastric epithelial cells (Fig. 6; refs. 12, 23, 33). In contrast, CLDN18.1 expression was detected at varying levels in several of these cells, with appreciable amounts observed in the lung (BSMC), kidney (HRE), and prostate (PrSC; Supplementary Fig. S11). The data showed that exposure to any of the normal cells failed to trigger TAC T-cell activation in contrast to CLDN18.2-positive control cells, as indicated by a lack of CD69 and cytokine expression (Fig. 6; Supplementary Table S9). Thus, these findings agree with our observations during the proteome study and support the supposition that TAC01-CLDN18.2 activity is specific to CLDN18.2.

## Discussion

T-cell activation induced by the natural TCR and endogenous cofactors, including the CD4/CD8 co-receptors, is orchestrated by a series of protein kinases and phosphatases that not only promote and amplify but also, in turn, antagonize TCR signaling (34). This awards T cells a degree of autoregulation and the capacity to adjust T-cell responses based on the environment (35). Accordingly, TCRs can react to low antigen densities that are well below the threshold of other synthetic receptors and can, conversely, taper intracellular signaling when encountering high antigen levels; for instance, in conjunction with regulatory domains within the cytoplasmic portion of an adjacent CD4 co-receptor (36, 37). Thus, the cytoplasmic tail of the CD4 co-receptor exerts important regulatory functions on TCR signaling and represents a key feature within the TAC receptor that is expected to leverage these properties and to maximize the therapeutic potential of T cells. Supporting evidence has been generated in multiple examples showing that TAC T cells are not burdened by tonic signaling or premature exhaustion and, instead, can lead to long-lasting T-cell persistence and antitumor activity (1, 3, 38). Similarly, CLDN18.2-TAC T cells did not exert any activity in the absence of the target antigen and—in the presence of tumor antigen—exhibited favorable pharmacology parameters, such as reacting to cancer cells expressing low CLDN18.2 levels, effective killing at low E:T ratios, clearance of tumor spheroids and *in vivo* xenografts, and long-lasting activity in recursive killing assays and tumor rechallenge experiments. Of note, CLDN18.2-TAC T cells demonstrated superior efficacy in a recursive killing assay compared with analogous CAR T cells. This corroborates our previous findings showing that HER2-TAC T cells outperformed CD28-based HER2-CAR T cells in a solid tumor mouse model with increased antitumor efficacy, reduced toxicity, and faster tumor infiltration (1). Thus, a limitation of the present study is the lack of an *in vivo* comparison using CLDN18.2-TAC T cells and CLDN18.2-CAR T cells which would further elucidate the potential superiority of TAC over conventional CAR designs. As observed in the spheroid assay, activated TAC T cells also led to an upregulation of PD-L1 in tumor cells, which suggests that a combined application with checkpoint inhibitors may increase the overall therapeutic capacity of TAC T cells.

Considering the functions naturally provided by the TCR and as briefly summarized above, several other chimeric receptor designs that utilize endogenous TCR signaling for improved therapeutic T-cell strategies are in development (39–42). These designs and mechanisms that induce TCR signaling differ substantially from the TAC receptor, however, making general inferences about this class of T-cell products difficult. For instance, TAC is unique in its structure by encompassing sequences of the CD4 co-receptor and interacts with the TCR noncovalently as a separate molecule.

A feature of the TAC previously highlighted is its safety advantage without compromising efficacy or the durability of antitumor responses (1). For instance, a HER2 antigen-binding domain used in a TAC T product proved antigen-specific, safe, and more efficacious whereas it induced toxic off-target effects in a second-generation CAR T product (1). Similarly, in this study, both the CLDN18.2-directed nanobody and CLDN18.2-TAC T cells were specific to CLDN18.2. TAC01-CLDN18.2 activation was strictly CLDN18.2-dependent even when cocultured with CLDN18.1-engineered control cells. This high level of specificity is critical given that the CLDN18.2-specific epitope, located in the first extracellular loop, only differs from its isoform 1 by a few amino acids. This finding was further supported by the observation that TAC01-CLDN18.2 did not show signs of activation in coculture with human primary cells endogenously expressing CLDN18.1.

CLDN18.2-TAC T cells cross-reacted with murine CLDN18.2 as both the human and murine isoforms share identical epitopes in the first extracellular loop. This allowed a close inspection of potential on-target/off-tumor effects in our mouse models, a concern raised during the development of other cell therapy products targeting tumor-associated antigens in solid tumors (43, 44). We had predicted that CLDN18.2 should be hidden within tight junctions in healthy tissues, thus allowing TAC01-CLDN18.2 to differentiate between healthy and tumor tissues. In line with this assumption, our data did not reveal any evidence of on-target/off-tumor side-effects. A *TAC*-specific ddPCR analysis of gastric tissue from TAC01-CLDN18.2–treated mice failed to identify an ongoing inflammatory event involving TAC T-cell proliferation as gastric *TAC* concentrations were steadily low and no higher than in other CLDN18.2-negative tissues. This agreed with merely transiently elevated serum cytokines during tumor regression as well as the histopathologic analysis and clinical observations (including body weight assessments) that were within the normal range. Thus, these data would support the thesis on tumor-selective targeting of CLDN18.2-positive tumors by TAC T cells. In a pathologic state in human subjects, however, it may be possible that the tumor affects the presentation of CLDN18.2 in normal cells, particularly in those that are located immediately adjacent to the tumor. Therefore, monitoring potential on-target/off-tumor effects in future clinical trials is recommended.

The emphasis is now on the clinical evaluation of TAC01-CLDN18.2 in a phase I/II study investigating the safety and efficacy in subjects with unresectable, locally advanced, or metastatic CLDN18.2-positive solid tumors (NCT05862324; ref. 17). This trial follows another clinical study evaluating a HER2-directed TAC T-cell product, TAC01-HER2, which previously demonstrated signs of efficacy and a manageable safety profile during a clinical phase I study in patients with advanced HER2-positive malignancies (NCT04727151; ref. 4). Therefore, our preclinical data presented herein combined with clinical data obtained from TAC01-HER2 support the further development of the CLDN18.2-directed TAC T therapy.

## Supplementary Material

Supplementary Figure 2 Supplementary Figure 2: TAC01-CLDN18.2 cytotoxicity against various solid tumor cells.

Supplementary Figure 3 Supplementary Figure 3: Head-to-head comparison of CLDN18.2-TAC T and CLDN18.2-CAR T cells in recursive killing assay.

Supplementary Figure 4 Supplementary Figure 4: In vivo efficacy of TAC01-CLDN18.2 across different donors in the OE19 tumor model.

Supplementary Figure 5 Supplementary Figure 5: Dose-dependent effects of TAC01-CLDN18.2 on OE19 tumor growth in NSG mice.

Supplementary Figure 6 Supplementary Figure 6: TAC01-CLDN18.2 biodistribution in NSG mice carrying OE19 tumor xenografts.

Supplementary Figure 7 Supplementary Figure 7: N87^CLDN18.2^ tumor growth during primary and secondary tumor challenge in DKO mice treated with non-transduced T cells (NTD).

Supplementary Figure 8 Supplementary Figure 8: Relative body weights of tumor-bearing NSG mice treated with TAC01-CLDN18.2.

Supplementary Figure 9 Supplementary Figure 9: Serum cytokine levels in mice treated with TAC01-CLDN18.2.

Supplementary Figure 10 Supplementary Figure 10: Histology of murine stomach tissues.

Supplementary Figure 11 Supplementary Figure 11: CLDN18.1 expression in healthy cell models.

Supplementary Tables Supplementary Tables 1-9

Supplementary Materials Supplementary Materials - Flow Cytometry

Supplementary Figure 1 Supplementary Figure 1: Specificity of recombinant hVH6-GFP protein in a protein array study encompassing >6,000 human membrane proteins.
