# Supplementary material for: Preclinical Development of T Cells Engineered to Express a T-Cell Antigen Coupler Targeting Claudin 18.2–Positive Solid Tumors
Source: Cancer Immunol Res. 2024 Oct 15;13(1):35–46. doi: 10.1158/2326-6066.CIR-24-0138 (PMC11712040; doi:10.1158/2326-6066.CIR-24-0138)
Supplement: Supplementary Figure 2 — TAC01-CLDN18.2 cytotoxicity against various solid tumor cells. [file cir-24-0138_supplementary_figure_2_supps2.docx]

**Supplementary Figure 2: TAC01-CLDN18.2 cytotoxicity against various solid tumor cells.**

TAC01-CLDN18.2 was cocultured at different E:T ratios with nGFP-engineered tumor cells naturally expressing CLDN18.2. Tumor cell growth was monitored via nGFP during live-cell imaging for 120 hours, and images were taken every 8 hrs. Each image was quantified, and the resulting time course is shown. Target cells alone were used as negative controls relative to TAC T-treated wells. Each data point represents the mean derived from 3 separate donors and 3 technical replicates. Error bars indicate standard deviation. The area under the curve (AUC) is calculated and is shown in the corresponding tables.
