# Supplementary material for: Preclinical Development of T Cells Engineered to Express a T-Cell Antigen Coupler Targeting Claudin 18.2–Positive Solid Tumors
Source: Cancer Immunol Res. 2024 Oct 15;13(1):35–46. doi: 10.1158/2326-6066.CIR-24-0138 (PMC11712040; doi:10.1158/2326-6066.CIR-24-0138)
Supplement: Supplementary Figure 3 — Head-to-head comparison of CLDN18.2-TAC T and CLDN18.2-CAR T cells in recursive killing assay. [file cir-24-0138_supplementary_figure_3_supps3.docx]

**Supplementary Figure 3: Head-to-head comparison of CLDN18.2-TAC T and CLDN18.2-CAR T cells in recursive killing assay.** T cells from two healthy donors were used to produce each 2 batches of TAC T and CD28-based 2^nd^-generation CAR T cells. Both TAC and CAR receptors encode the hVH6 CLDN18.2-binding domain. TAC T and CAR T cells were cocultured with N87^CLDN18.2/GFP^ target cells at an E:T ratio of 3:1 in triplicates. All engineered cells were adjusted to the same transduction levels by adding NTD cells of the matching donor as required. N87^CLDN18.2/GFP^ target cells were seeded one day prior to T cells addition to ensure target cells adherence. During the coculture, GFP fluorescence was measured in triplicates at the beginning and at the end of the round as an indicator of target cell viability (**A**). Each round alternated between 3 or 4 days. At the end of each round, T cells were harvested, pooled, and counted (**B**). At select time points, T cells were also phenotyped for determining proportions of CD4/8 and exhausted T cells (**C**, PD-1 alone; **D**, PD-1/LAG3/TIM3 signature). Then, a new coculture was set up by adding harvested T cells to pre-plated target cells at a 3:1 ratio. Individual data per donor are shown.
