# Supplementary material for: Preclinical Development of T Cells Engineered to Express a T-Cell Antigen Coupler Targeting Claudin 18.2–Positive Solid Tumors
Source: Cancer Immunol Res. 2024 Oct 15;13(1):35–46. doi: 10.1158/2326-6066.CIR-24-0138 (PMC11712040; doi:10.1158/2326-6066.CIR-24-0138)
Supplement: Supplementary Figure 4 — In vivo efficacy of TAC01-CLDN18.2 across different donors in the OE19 tumor model. [file cir-24-0138_supplementary_figure_4_supps4.docx]

**Supplementary Figure 4: In vivo efficacy of TAC01-CLDN18.2 across different donors in the OE19 tumor model.**

Female NSG mice bearing OE19 solid tumors were treated with either 6 x 10^6^ CLDN18.2-TAC T cells (n=8 per group) manufactured using 4 different donors, or the corresponding NTD T cells (n= 4-8 per group) on day 0. An untreated (NT) group of mice was included as a negative control (n=4-5).
