# Supplementary material for: Preclinical Development of T Cells Engineered to Express a T-Cell Antigen Coupler Targeting Claudin 18.2–Positive Solid Tumors
Source: Cancer Immunol Res. 2024 Oct 15;13(1):35–46. doi: 10.1158/2326-6066.CIR-24-0138 (PMC11712040; doi:10.1158/2326-6066.CIR-24-0138)
Supplement: Supplementary Figure 5 — Dose-dependent effects of TAC01-CLDN18.2 on OE19 tumor growth in NSG mice. [file cir-24-0138_supplementary_figure_5_supps5.docx]

**Supplementary Figure 5: Dose-dependent effects of TAC01-CLDN18.2 on OE19 tumor growth in NSG mice.**

Female NSG mice bearing OE19 solid tumors were treated with different dose levels of CLDN18.2-TAC, ranging from 0.5 x 10^6^ – 6 x 10^6^ TAC T cells or the corresponding number of NTD T cells on day 0 via tail vein injection (n= 6 per group). An untreated (NT) group of mice was included as a negative control (n=5).
