# Supplementary material for: Preclinical Development of T Cells Engineered to Express a T-Cell Antigen Coupler Targeting Claudin 18.2–Positive Solid Tumors
Source: Cancer Immunol Res. 2024 Oct 15;13(1):35–46. doi: 10.1158/2326-6066.CIR-24-0138 (PMC11712040; doi:10.1158/2326-6066.CIR-24-0138)
Supplement: Supplementary Figure 6 — TAC01-CLDN18.2 biodistribution in NSG mice carrying OE19 tumor xenografts. [file cir-24-0138_supplementary_figure_6_supps6.docx]

**Supplementary Figure 6: TAC01-CLDN18.2 biodistribution in NSG mice carrying OE19 tumor xenografts.**

*TAC* copies in various tissues at different timepoints quantified by ddPCR in OE19 tumor-bearing mice treated with TAC01-CLDN18.2. Data are represented as the mean ± standard deviation (n=6 per timepoint; 3 female, 3 male). Dotted lines indicate the LLOQ and LLOD of the ddPCR assay.
