# Supplementary material for: Preclinical Development of T Cells Engineered to Express a T-Cell Antigen Coupler Targeting Claudin 18.2–Positive Solid Tumors
Source: Cancer Immunol Res. 2024 Oct 15;13(1):35–46. doi: 10.1158/2326-6066.CIR-24-0138 (PMC11712040; doi:10.1158/2326-6066.CIR-24-0138)
Supplement: Supplementary Figure 7 — N87CLDN18.2 tumor growth during primary and secondary tumor challenge in DKO mice treated with non-transduced T cells (NTD). [file cir-24-0138_supplementary_figure_7_supps7.docx]

**Supplementary Figure 7: N87^CLDN18.2^ tumor growth during primary and secondary tumor challenge in DKO mice treated with non-transduced T cells (NTD).**

NSG/MHC-DKO mice engrafted with N87^CLDN18.2^ tumor xenografts were treated once with NTD T cells (day 0). Mice were rechallenged with N87^CLDN18.2^ tumor cells given in the opposing hind flank (dotted line, day 28) of the same mice. **A.** Individual tumor volumes of mice on the primary site. **B.** Individual tumor volumes of secondary tumor challenge on the opposite flank. Mice are the same as numbered in A.
