# Supplementary material for: Preclinical Development of T Cells Engineered to Express a T-Cell Antigen Coupler Targeting Claudin 18.2–Positive Solid Tumors
Source: Cancer Immunol Res. 2024 Oct 15;13(1):35–46. doi: 10.1158/2326-6066.CIR-24-0138 (PMC11712040; doi:10.1158/2326-6066.CIR-24-0138)
Supplement: Supplementary Figure 8 — Relative body weights of tumor-bearing NSG mice treated with TAC01-CLDN18.2. [file cir-24-0138_supplementary_figure_8_supps8.docx]

**Supplementary Figure 8: Relative body weights of tumor-bearing NSG mice treated with TAC01-CLDN18.2.**

Relative body weights of tumor-bearing mice relative to day 0 (100%), shown individually. The dotted line represents the ethical threshold of acceptable/tolerable weight loss. Corresponding tumor volumes are shown in **Figure 5A**.
