# Supplementary material for: Preclinical Development of T Cells Engineered to Express a T-Cell Antigen Coupler Targeting Claudin 18.2–Positive Solid Tumors
Source: Cancer Immunol Res. 2024 Oct 15;13(1):35–46. doi: 10.1158/2326-6066.CIR-24-0138 (PMC11712040; doi:10.1158/2326-6066.CIR-24-0138)
Supplement: Supplementary Figure 9 — Serum cytokine levels in mice treated with TAC01-CLDN18.2. [file cir-24-0138_supplementary_figure_9_supps9.docx]

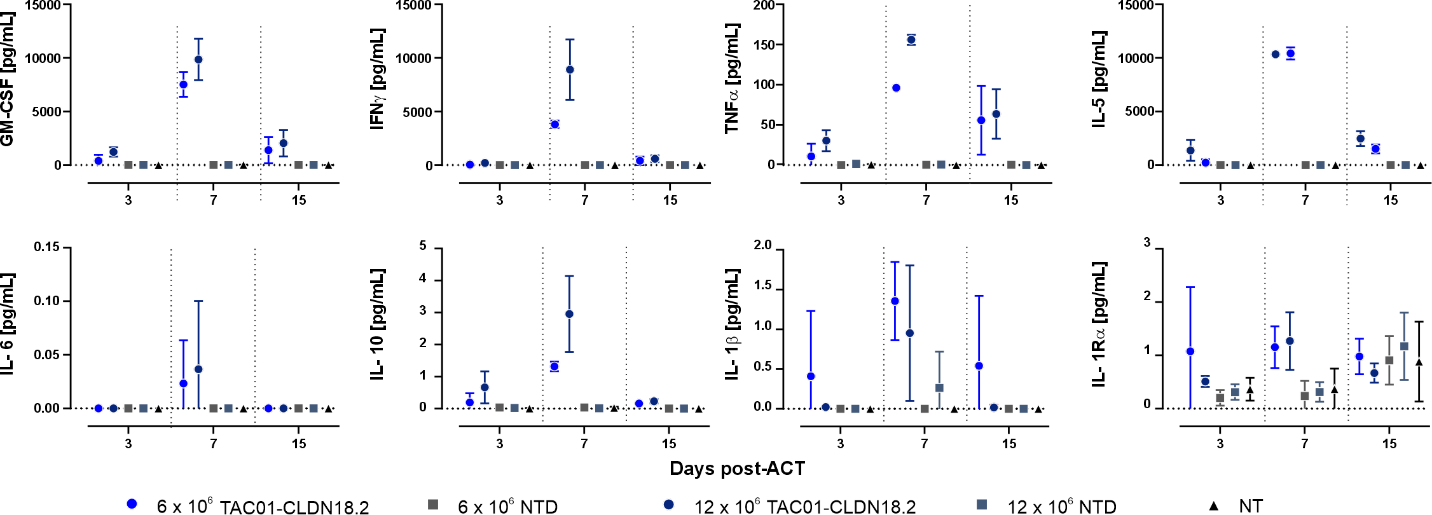


**Supplementary Figure 9: Serum cytokine levels in mice treated with TAC01-CLDN18.2.**

MHC DKO mice carrying established subcutaneous OE19 tumor xenografts were intravenously administered a single dose of CLDN18.2-TAC T cells at a dose level of 6 x 10^6^ or 12 x 10^6^ TAC T. Untreated animals (NT) and animals treated with non-transduced T cells (NTD) served as controls. Serum samples were collected on time points as indicated and evaluated for cytokine levels (Eve Biotechnologies, Vancouver; days 3 & 7:, n=3/grp; day 15: n=4/grp). A statistical analysis is provided in **Suppl. Table 8**.
