# Supplementary material for: Preclinical Development of T Cells Engineered to Express a T-Cell Antigen Coupler Targeting Claudin 18.2–Positive Solid Tumors
Source: Cancer Immunol Res. 2024 Oct 15;13(1):35–46. doi: 10.1158/2326-6066.CIR-24-0138 (PMC11712040; doi:10.1158/2326-6066.CIR-24-0138)
Supplement: Supplementary Figure 10 — Histology of murine stomach tissues. [file cir-24-0138_supplementary_figure_10_supps10.docx]

**
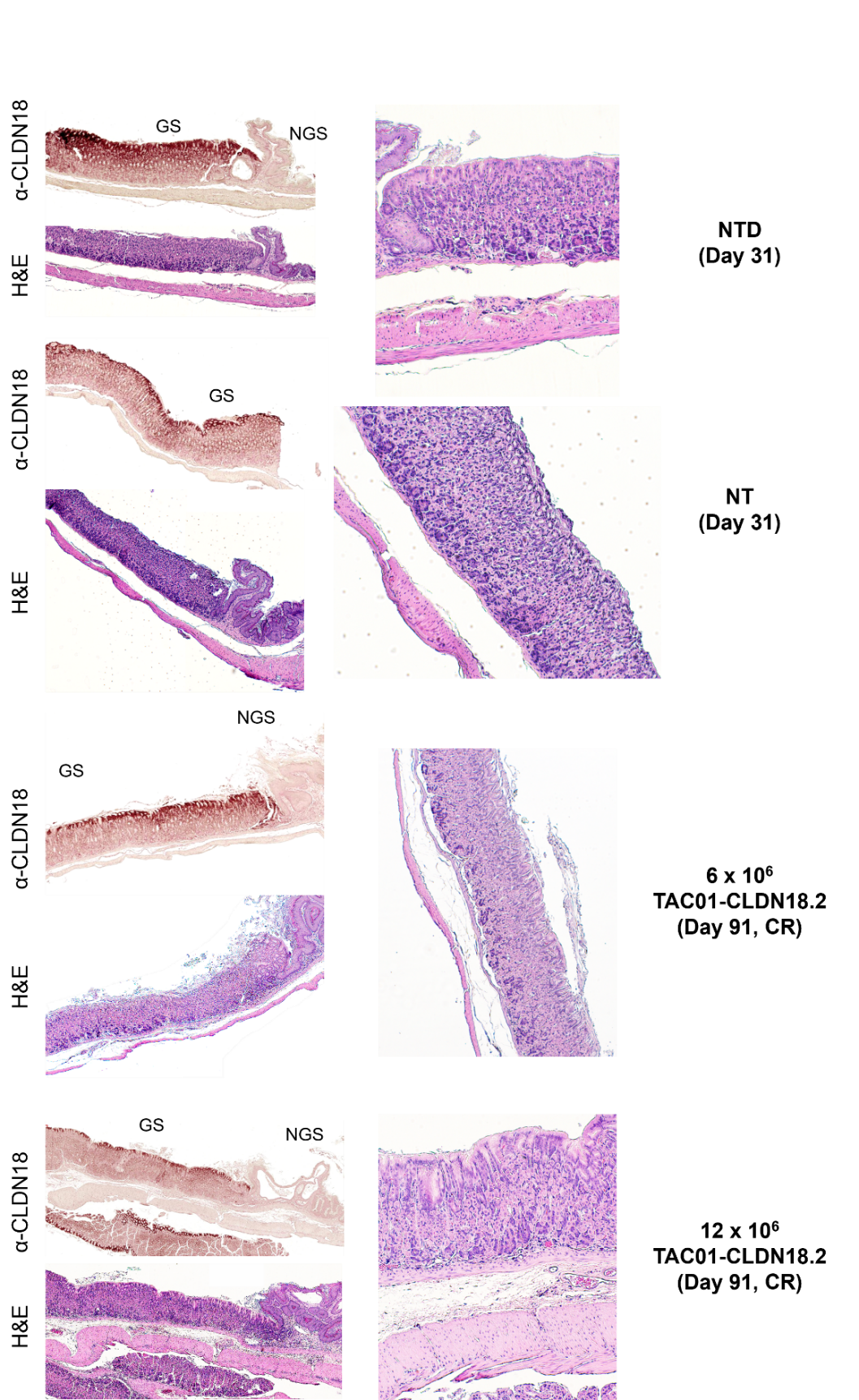
**

**Supplementary Figure 10: Histology of murine stomach tissues.**

Stomach tissue from MHC DKO mice bearing OE19 tumors and treated with TAC01-CLDN18.2 were stained with a pan-Claudin18 antibody (clone 34H14L15) and H&E. CLDN18 staining was found to be restricted to the glandular stomach. CR = complete response; GS = glandular stomach; NGS = non-glandular stomach.
