# Supplementary material for: Preclinical Development of T Cells Engineered to Express a T-Cell Antigen Coupler Targeting Claudin 18.2–Positive Solid Tumors
Source: Cancer Immunol Res. 2024 Oct 15;13(1):35–46. doi: 10.1158/2326-6066.CIR-24-0138 (PMC11712040; doi:10.1158/2326-6066.CIR-24-0138)
Supplement: Supplementary Figure 11 — CLDN18.1 expression in healthy cell models. [file cir-24-0138_supplementary_figure_11_supps11.docx]

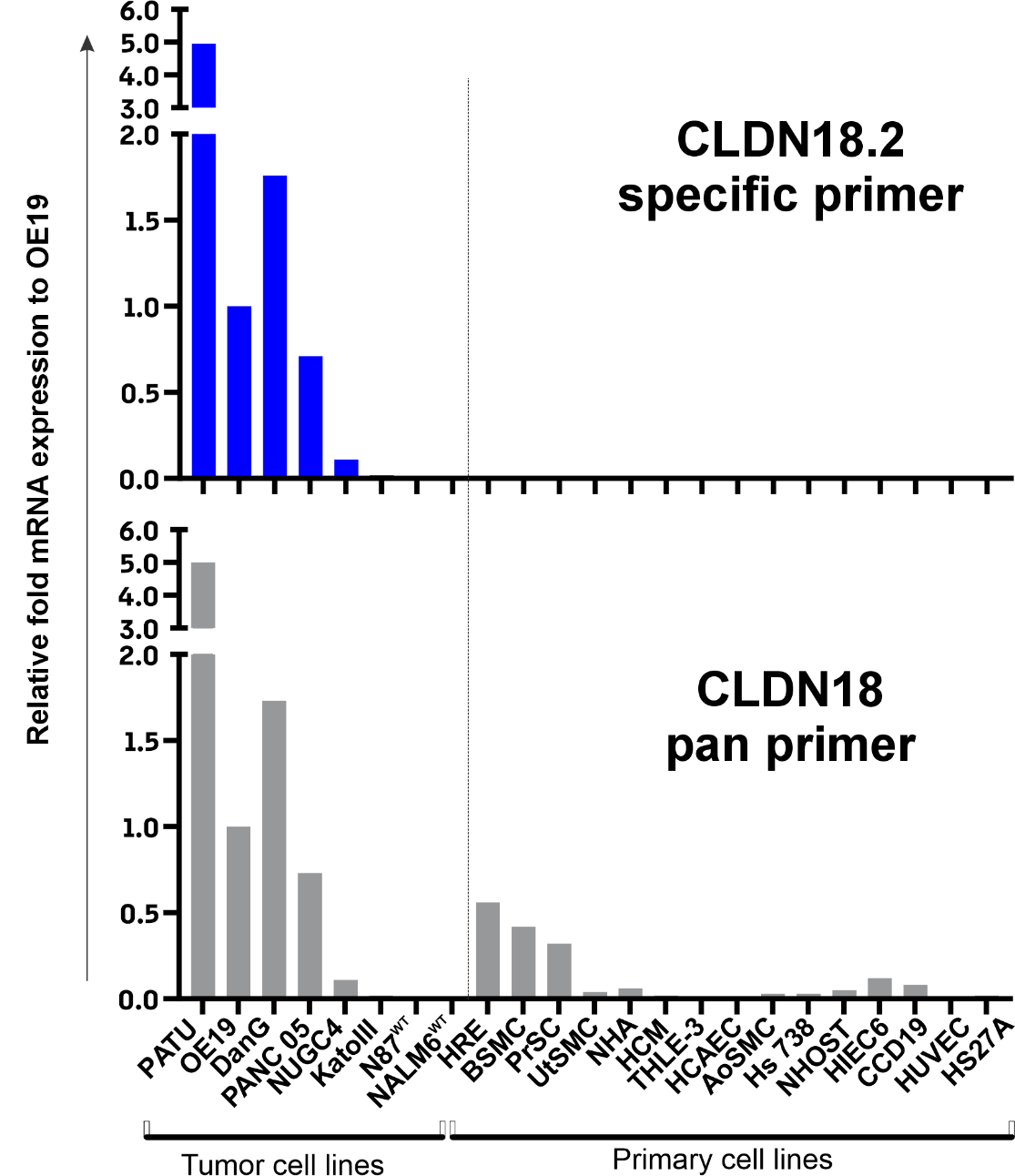


**Supplementary Figure 11: CLDN18.1 expression in healthy cell models.**

CLDN18.1 expression was assessed indirectly. The expression of CLDN18.2 using a CLDN18.2-specific primer set was compared to the expression of total CLDN18 (CLDN18.1 and CLDN18.2) measured by a pan-specific primer set. All data was normalized to OE19. As expected, cell lines that are positive for CLDN18.2 are also positive for the pan specific primer set, with the same ratios. Cell lines that only show positive signal with the CLD18 pan primer are considered CLDN18.1-positive.
