# Supplementary material for: Preclinical Development of T Cells Engineered to Express a T-Cell Antigen Coupler Targeting Claudin 18.2–Positive Solid Tumors
Source: Cancer Immunol Res. 2024 Oct 15;13(1):35–46. doi: 10.1158/2326-6066.CIR-24-0138 (PMC11712040; doi:10.1158/2326-6066.CIR-24-0138)

Table of Contents

Flow Cytometry Dot Plots Corresponding to Data Shown in Figures

Page	Flow Cytometry Analysis	Corresponding Figure
2	General gating strategy	
3	Expression of memory markers in TAC01-CLDN18.2	Figure 1B
4	TAC T cell activation by $N87^{hCLDN18.2}$ and $N87^{hCLDN18.1}$	Figure 1C
5	TAC T cell activation by $N87^{hCLDN18.2}$ and $N87^{mCLDN18.2}$	Figure 1D
6	TAC T cell activation (CD69)	Figure 2B
7-9	TAC T cell activation (cytokines)	Figure 2B
10	TAC T cell proliferation	Figure 2C
11	Example plots - TAC T cell activation by normal cells (CD69)	Figure 6
12-14	Example plots - TAC T cell activation by normal cells (cytokines)	Figure 6

General gating strategy in live cells

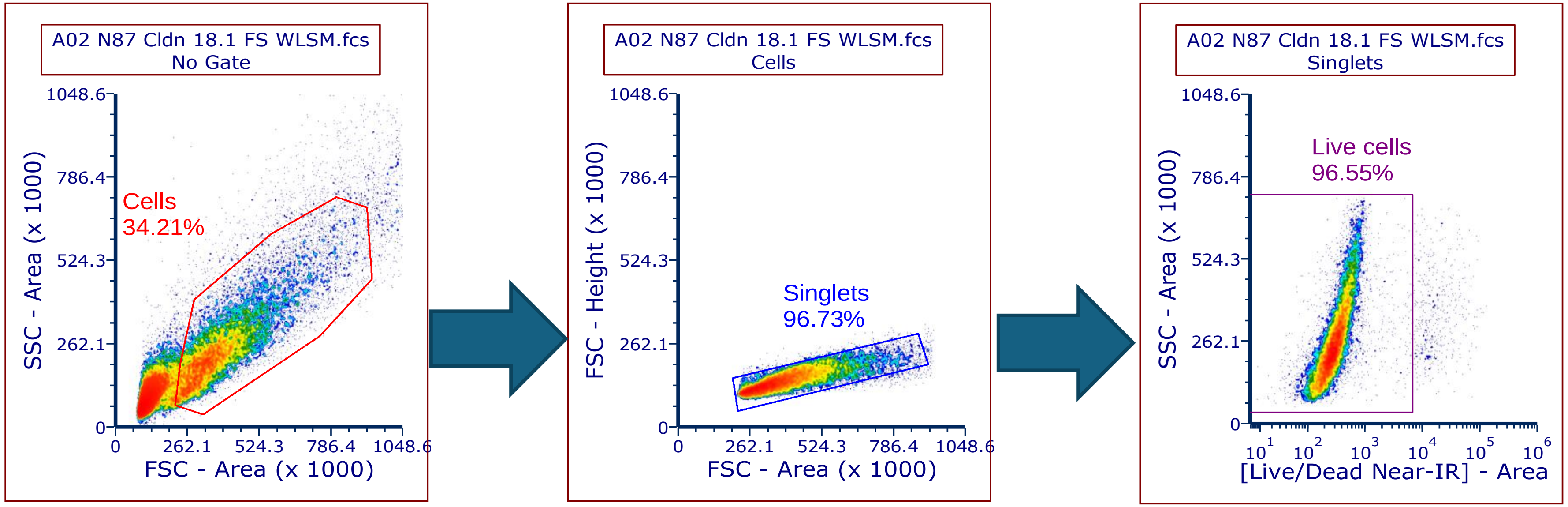

Figure 1B (right): Expression of memory markers in TAC01-CLDN18.2

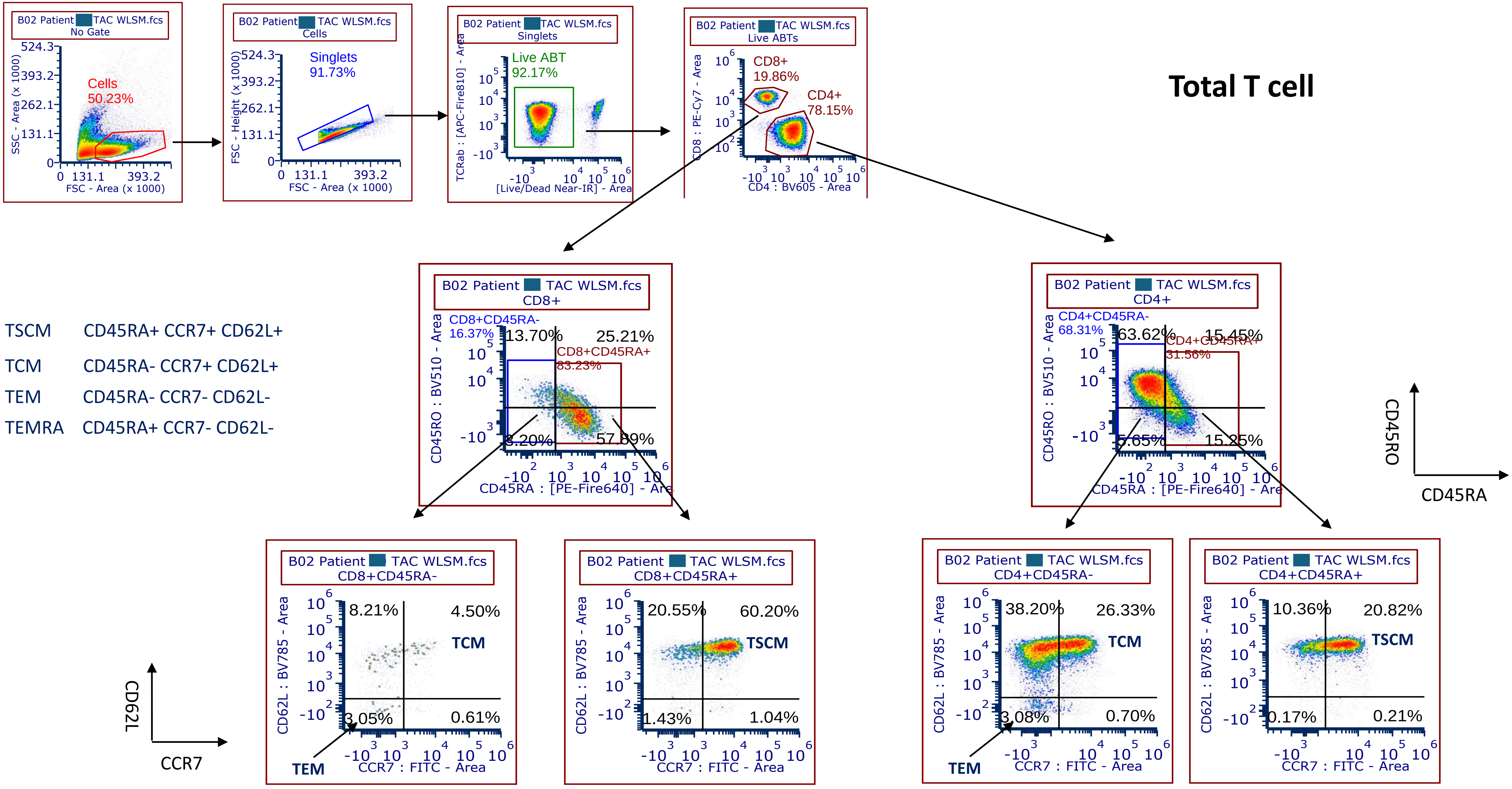

Figure 1C: TAC T cell activation by N87 expressing human CLDN18.1 and CLDN18.2

(A) Ectopic CLDN18.2 and CLDN18.1 expression in N87

(B) TAC T cell activation in coculture with various N87 cells as measured by surface CD69

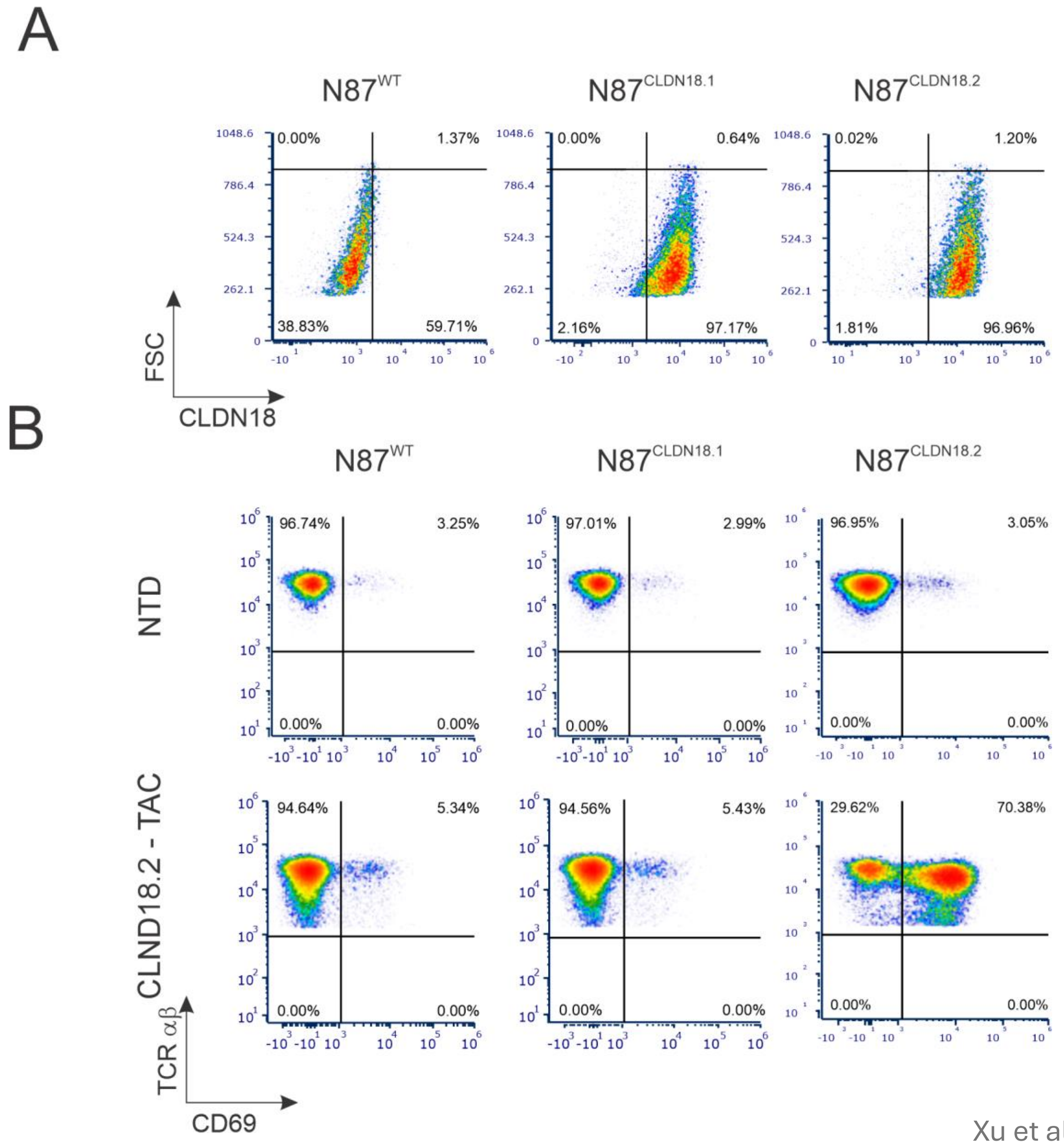

Figure 1D

TAC T cell activation by N87 cells ectopically expressing human CLDN18.2 vs murine CLDN18.2

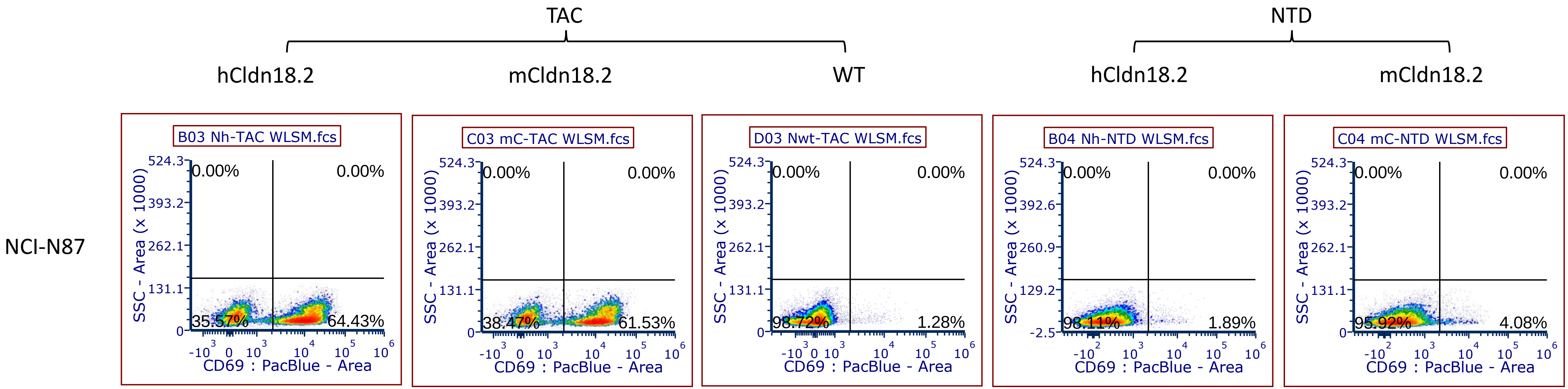

Figure 2B

CD69

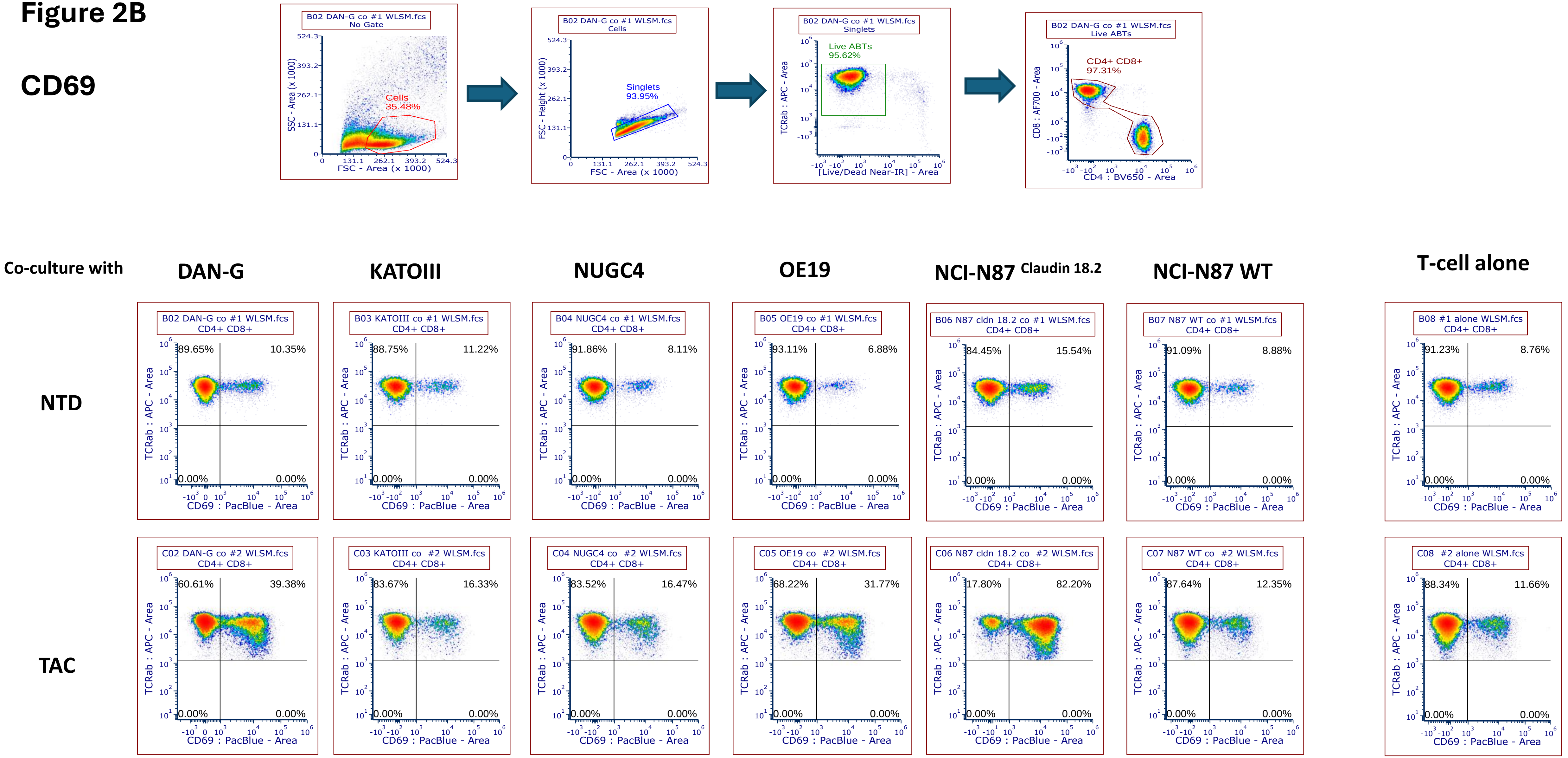

Figure 2B

IFNγ

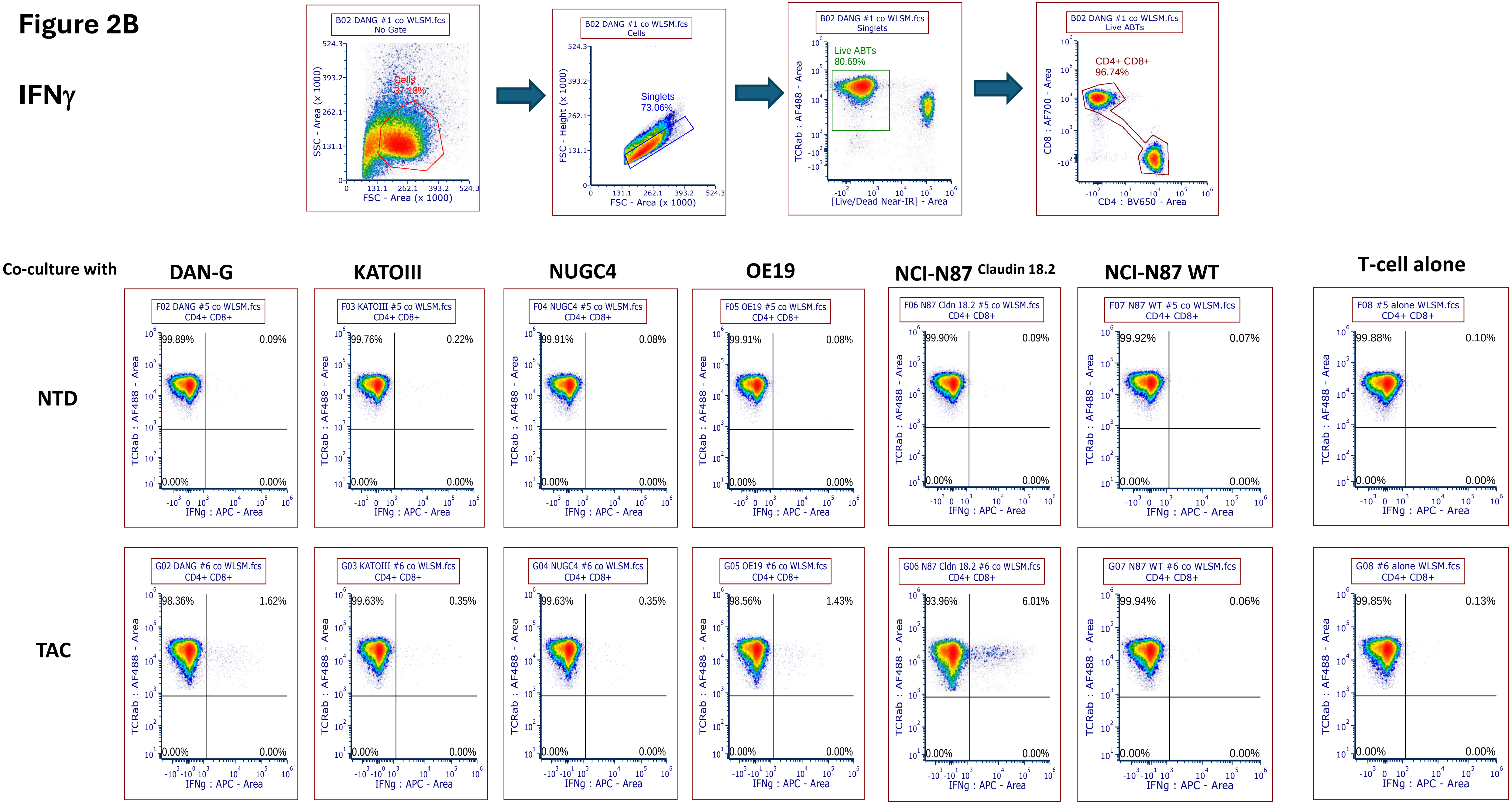

Figure 2B

TNFα

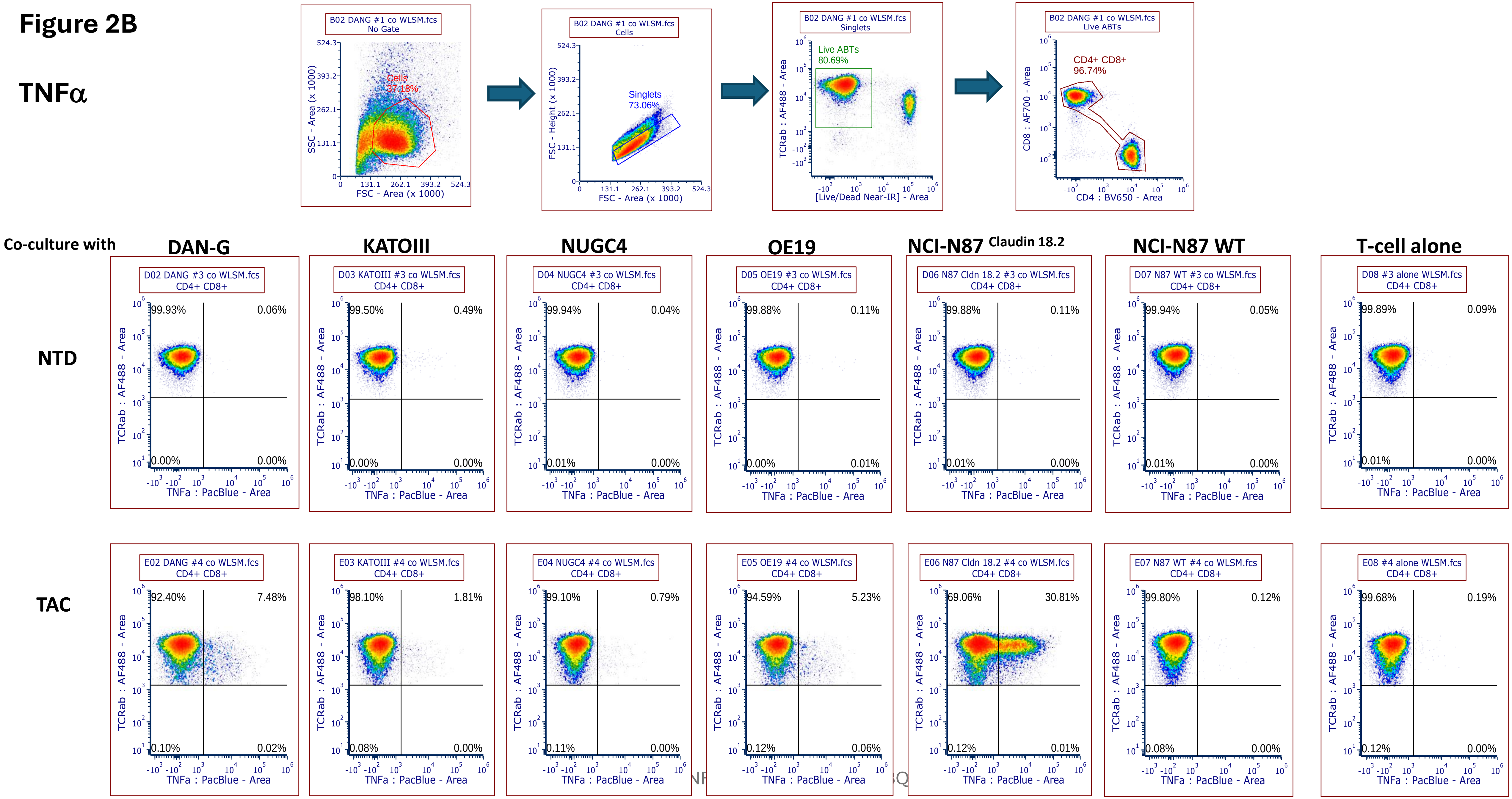

Figure 2B

IL2

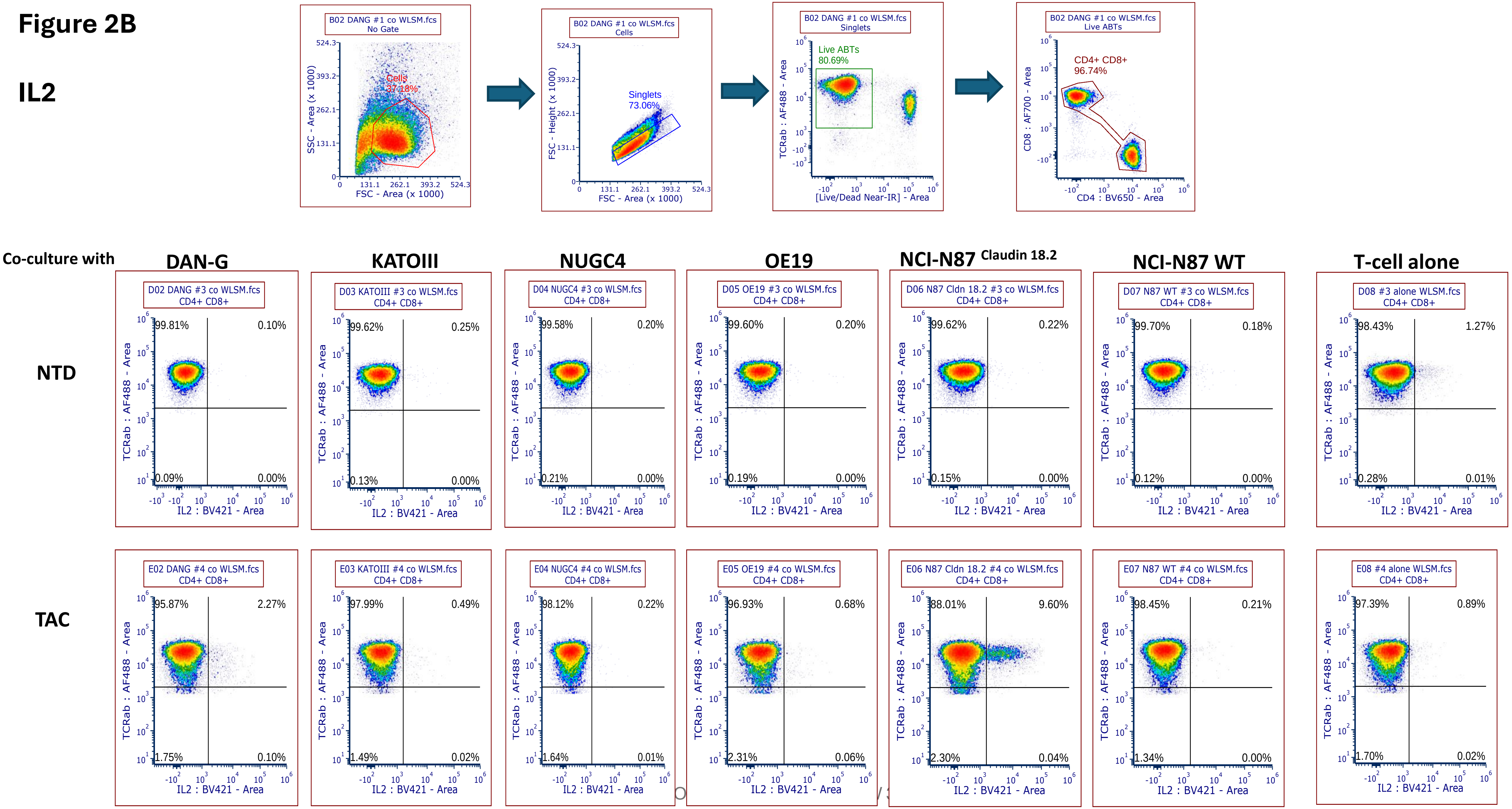

Figure 2C

Division

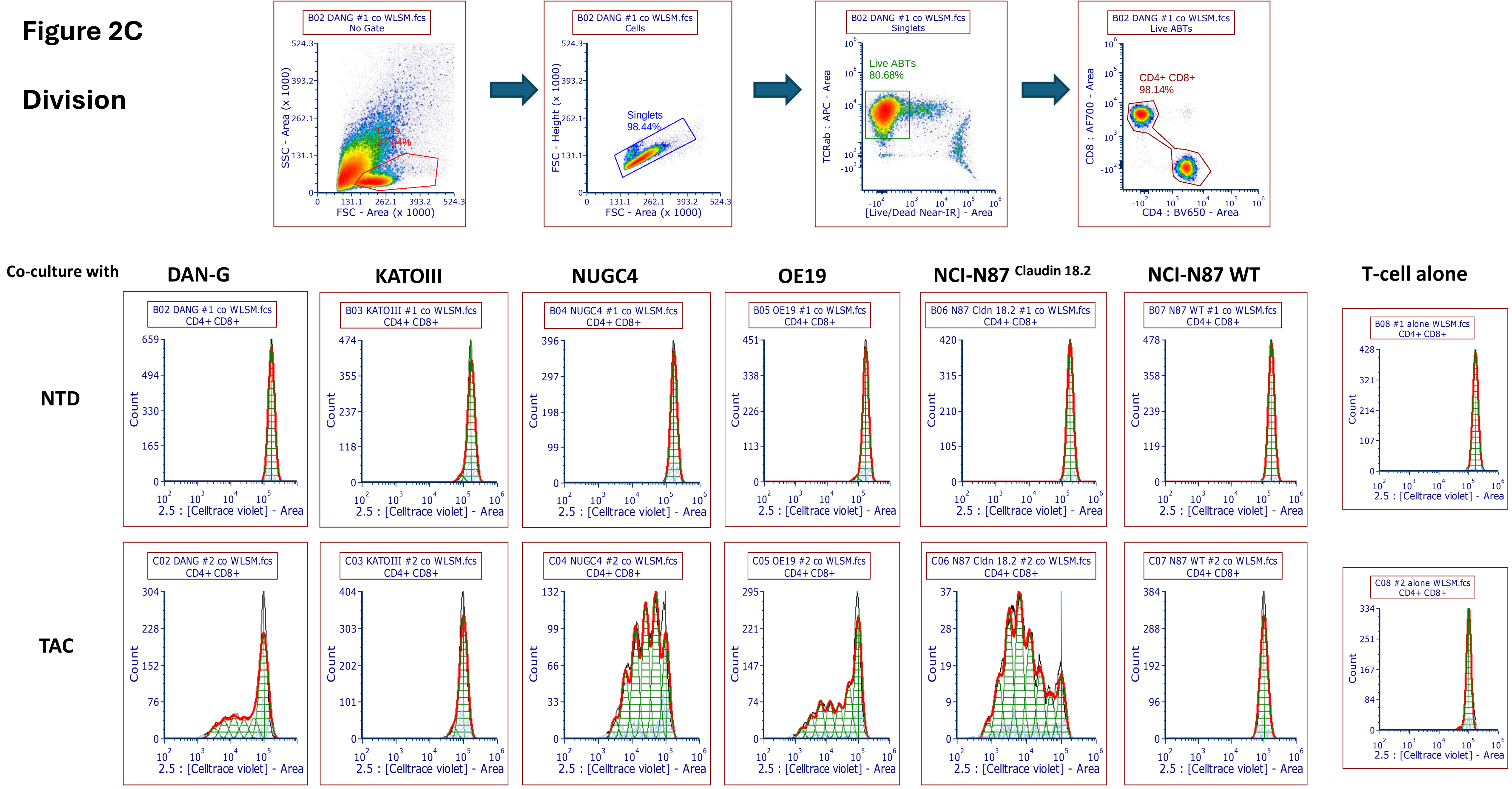

Figure 6 (middle panel)

CD69

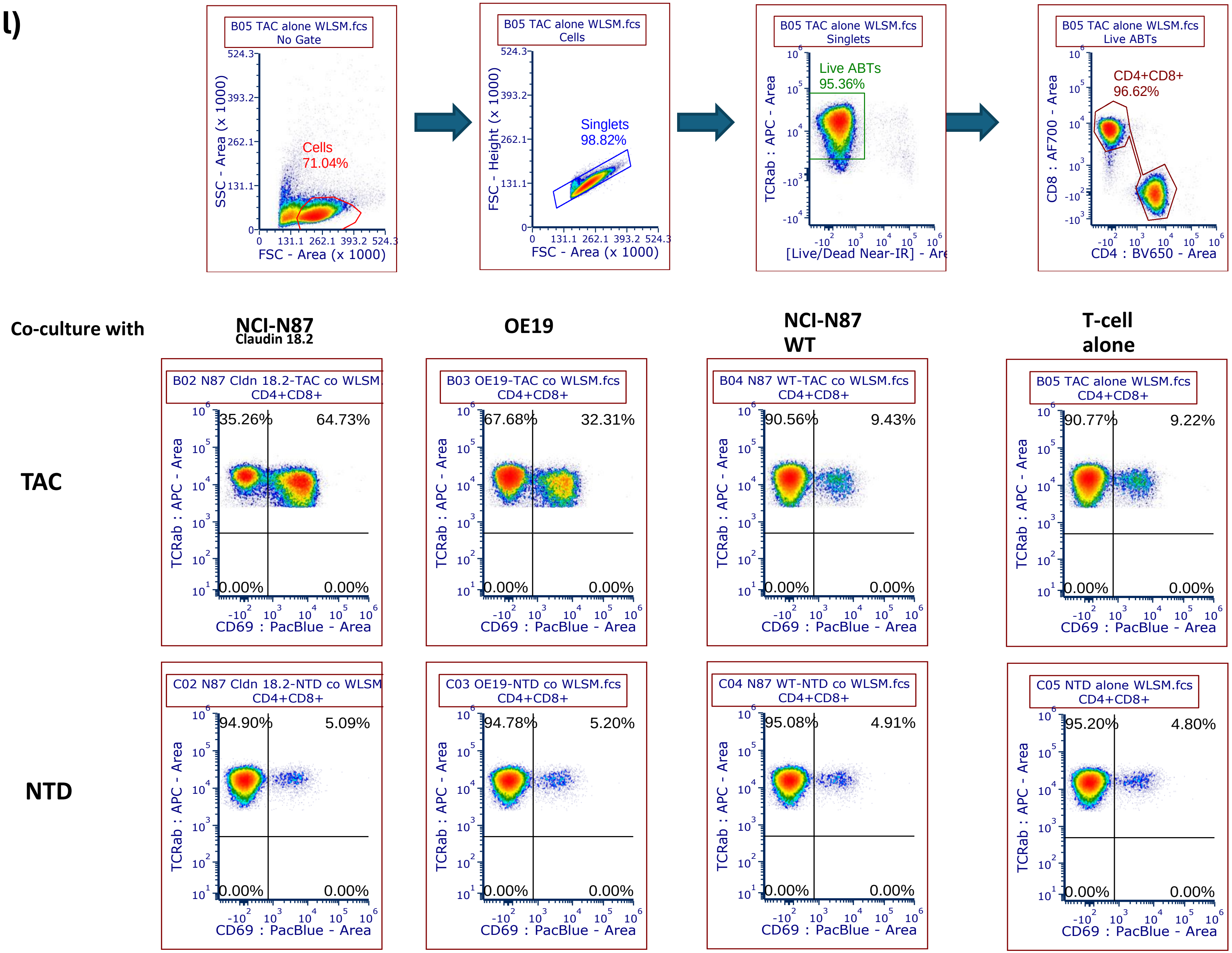

Figure 6 (bottom panel)

TNFα

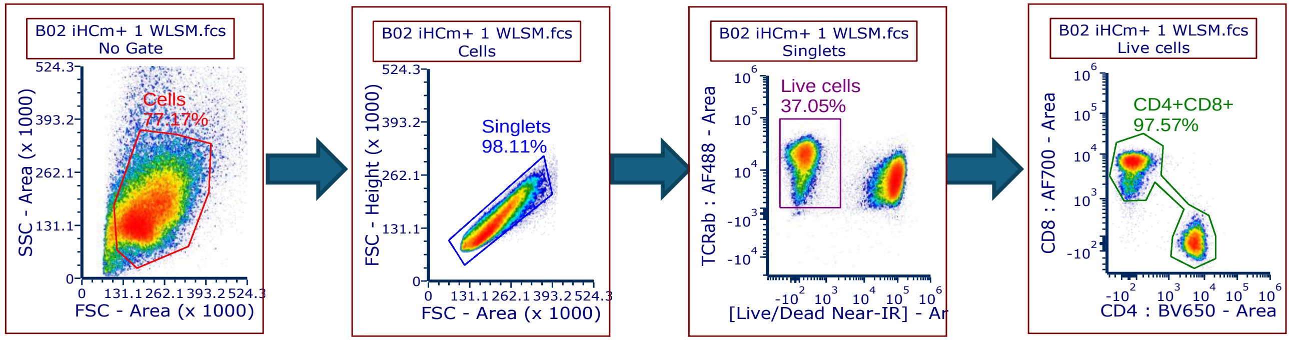

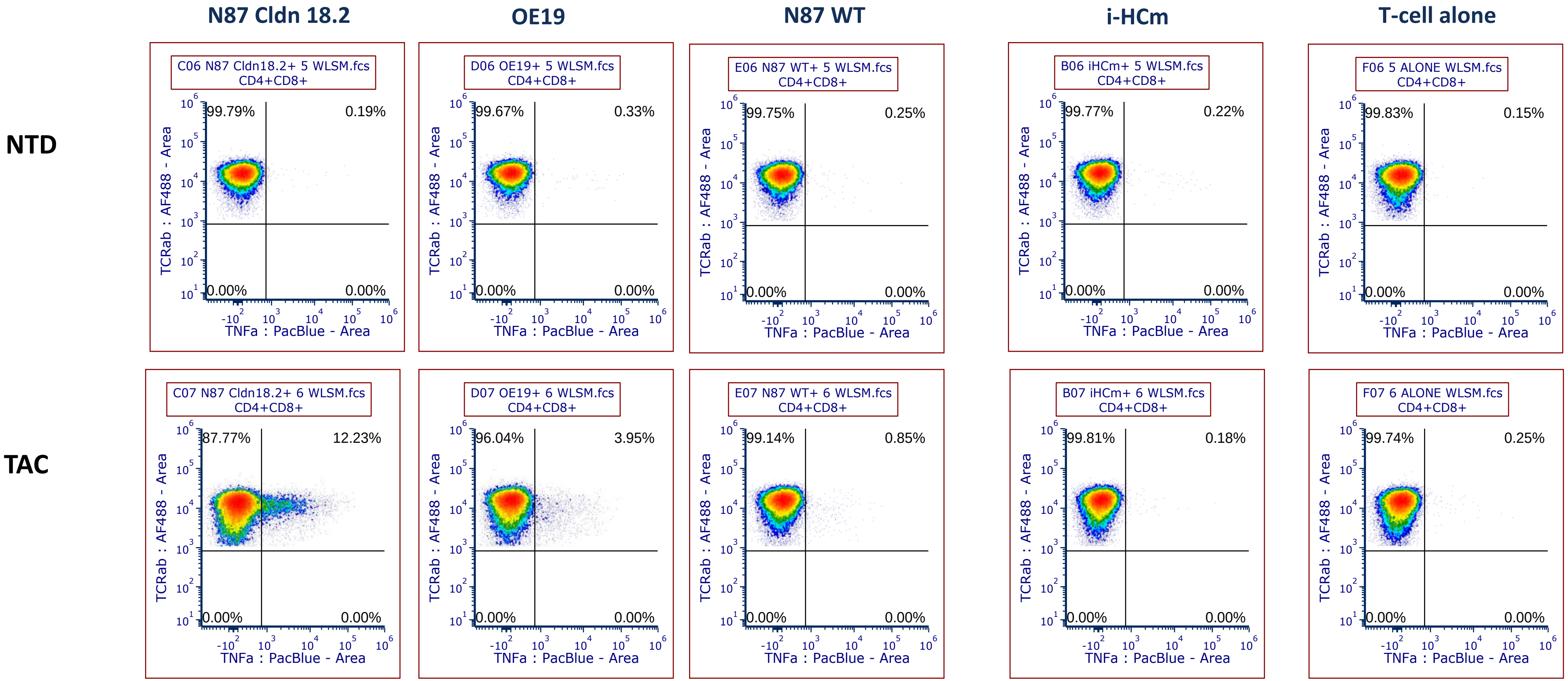

Figure 6 (bottom panel)

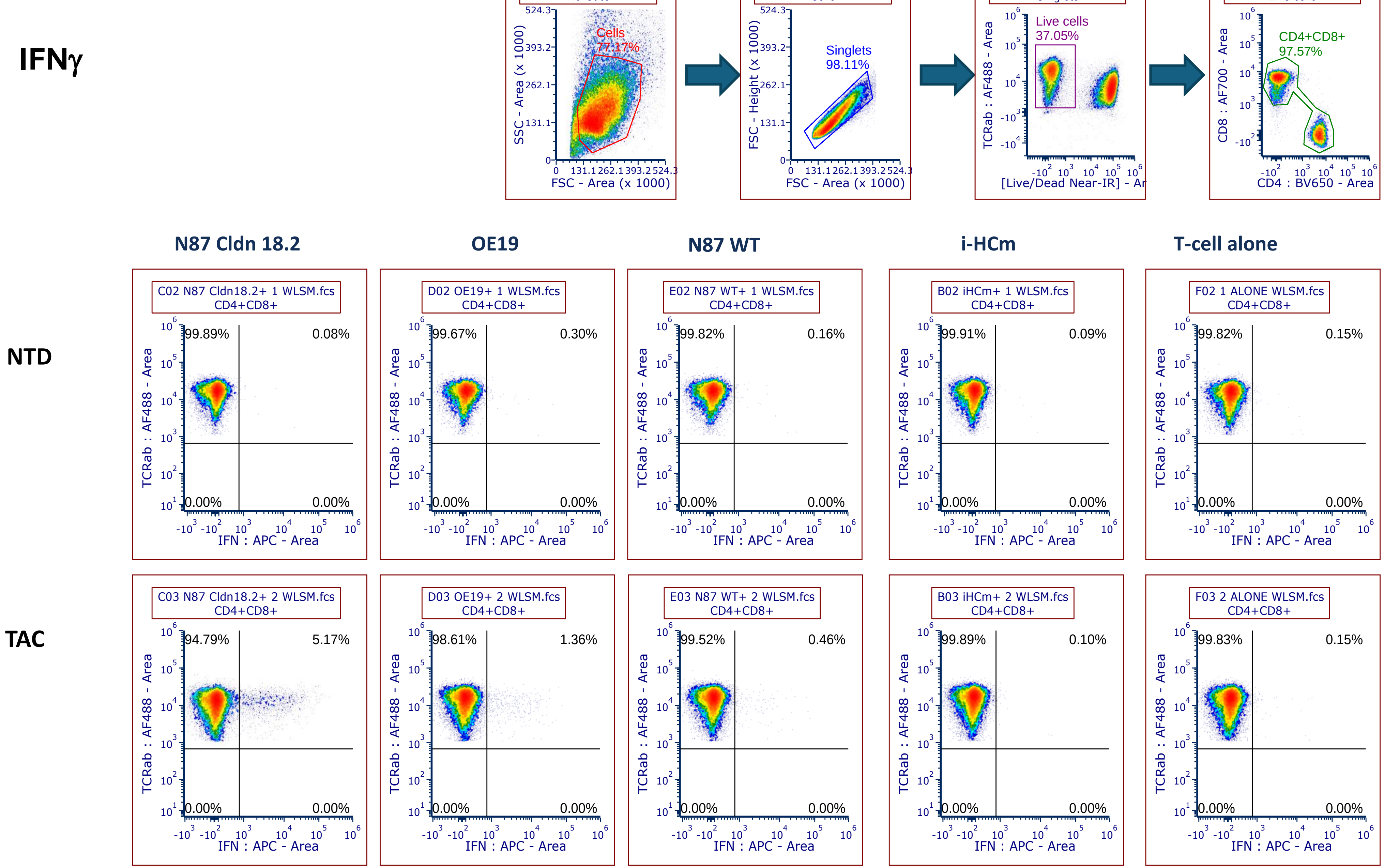

Figure 6 (bottom panel)

IL2

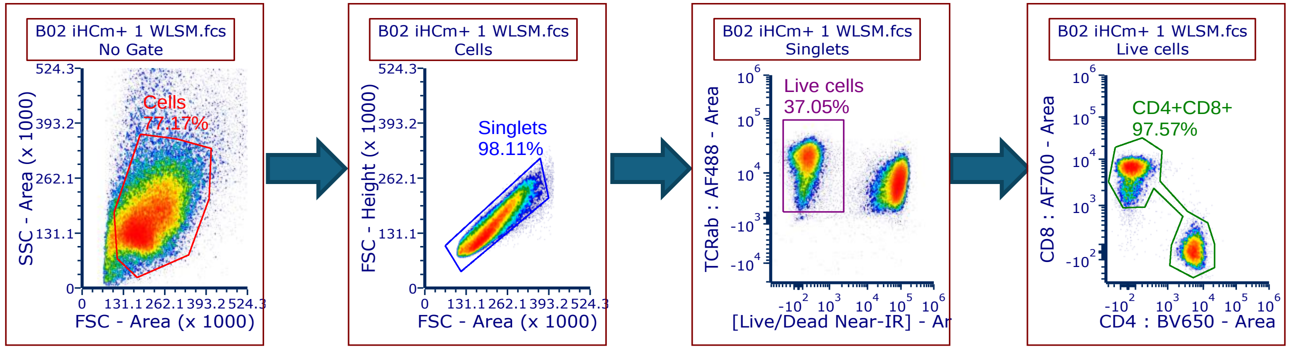

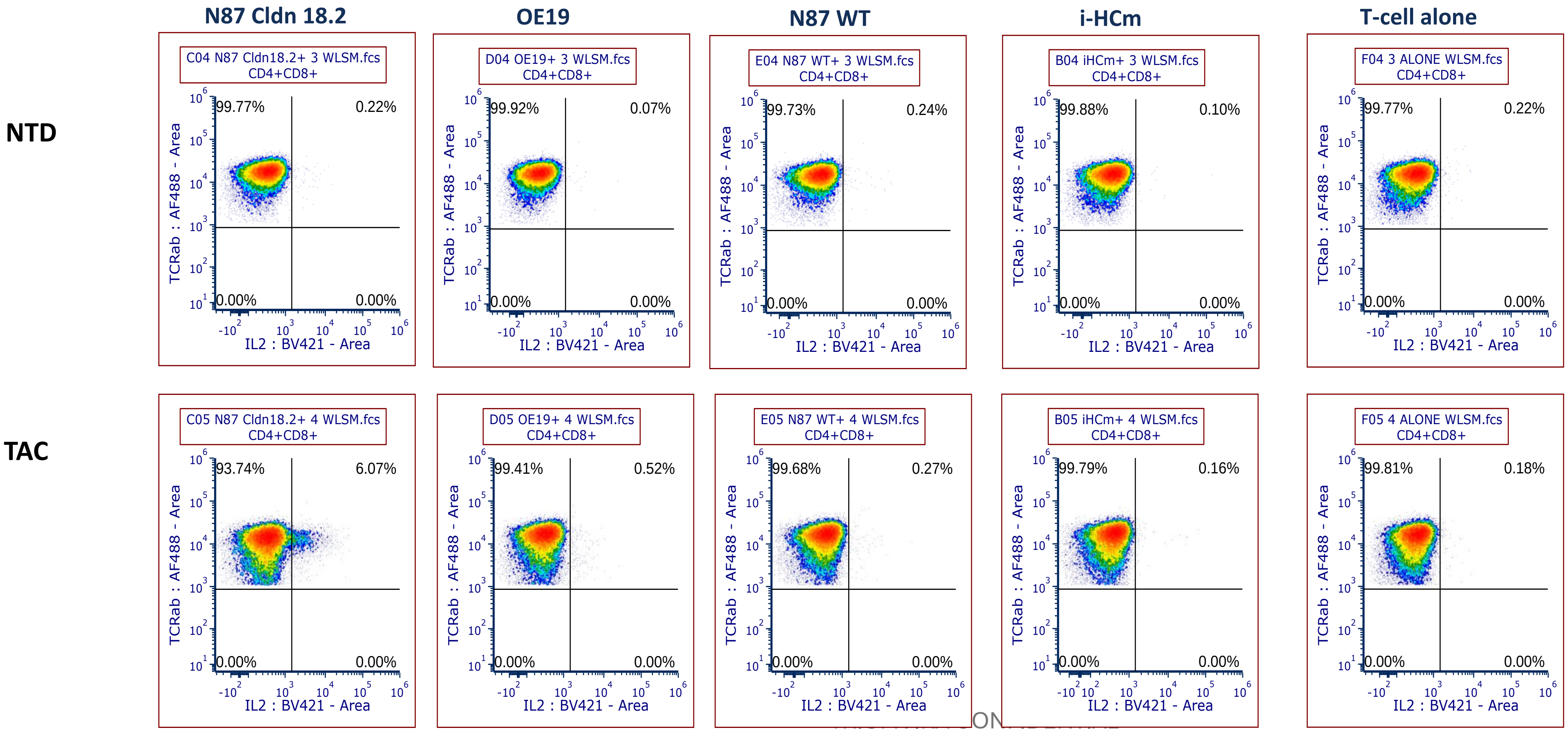

Supplement: Supplementary Materials — Flow Cytometry [file cir-24-0138_supplementary_materials_suppsd.pdf]
