# Supplementary material for: Preclinical Development of T Cells Engineered to Express a T-Cell Antigen Coupler Targeting Claudin 18.2–Positive Solid Tumors
Source: Cancer Immunol Res. 2024 Oct 15;13(1):35–46. doi: 10.1158/2326-6066.CIR-24-0138 (PMC11712040; doi:10.1158/2326-6066.CIR-24-0138)
Supplement: Supplementary Figure 1 — Specificity of recombinant hVH6-GFP protein in a protein array study encompassing >6,000 human membrane proteins. [file cir-24-0138_supplementary_figure_1_supps1.docx]

**Supplementary Figure 1: Specificity of recombinant hVH6-GFP protein in a protein array study encompassing >6000 human membrane proteins**. Top left: Titration of hVH6-GFP protein in a binding assay using HEK293T cells transfected with empty vector or vector encoding human CLDN18.2. Bound ligand was quantified by flow cytometry. Top right: hVH6-GFP (16 mg/mL) in proteome study. Binding data of hVH6-GFP done in replicates across the protein library including controls were established via a fluorescently labeled secondary antibody, transformed, and plotted as a unitless, ranked, non-linear score for target binding. Test ligand interactions with any targets identified by MPA screening were confirmed in a second flow cytometry experiment using serial dilutions of the test antibody, and the target identity was confirmed by sequencing. Bottom: Comparative binding of CLDN family members in the proteome study.
